# Chromosome evolution in Cophomantini (Amphibia, Anura, Hylinae)

**DOI:** 10.1371/journal.pone.0192861

**Published:** 2018-02-14

**Authors:** Juan M. Ferro, Dario E. Cardozo, Pablo Suárez, Juan M. Boeris, Ailin Blasco-Zúñiga, Gastón Barbero, Anderson Gomes, Thiago Gazoni, William Costa, Cleusa Y. Nagamachi, Miryan Rivera, Patricia P. Parise-Maltempi, John E. Wiley, Julio C. Pieczarka, Celio F. B. Haddad, Julián Faivovich, Diego Baldo

**Affiliations:** 1 Laboratorio de Genética Evolutiva, Instituto de Biología Subtropical (CONICET-UNaM), Facultad de Ciencias Exactas Químicas y Naturales, Universidad Nacional de Misiones, Posadas, Misiones, Argentina; 2 Instituto de Biología Subtropical (CONICET-UNaM), Puerto Iguazú, Misiones, Argentina; 3 Laboratorio de Investigación en Citogenética y Biomoléculas de Anfibios (LICBA), Centro de Investigación para la Salud en América Latina (CISeAL), Pontificia Universidad Católica del Ecuador, Quito, Ecuador; 4 Centro de Estudios Biomédicos, Biotecnológicos, Ambientales y Diagnóstico, Universidad Maimónides, CONICET, Buenos Aires, Argentina; 5 Instituto Federal de Educação, Ciência e Tecnologia do Pará, Abaetetuba, Pará, Brazil; 6 Departamento de Biologia, Instituto de Biociências, UNESP – Univ. Estadual Paulista, Campus de Rio Claro, São Paulo, Brasil; 7 Departamento de Biologia Estrutural e Funcional, Instituto de Biologia, UNICAMP – Univ. Estadual de Campinas, Campinas, Brasil; 8 Laboratório de Citogenética, Centro de Estudos Avançados da Biodiversidade, Instituto de Ciências Biológicas, Universidade Federal do Pará, Belém, Pará, Brasil; 9 The Brody School of Medicine, East Carolina University, Greenville, North Carolina, United States of America; 10 Departamento de Zoologia e Centro de Aquicultura, Instituto de Biociências, UNESP – Univ. Estadual Paulista, Campus de Rio Claro, São Paulo, Brasil; 11 División Herpetología, Museo Argentino de Ciencias Naturales “Bernardino Rivadavia”—CONICET, Buenos Aires, Argentina; 12 Departamento de Biodiversidad y Biología Experimental, Facultad de Ciencias Exactas y Naturales, Universidad de Buenos Aires, Buenos Aires, Argentina; University of Arkansas, UNITED STATES

## Abstract

The hylid tribe Cophomantini is a diverse clade of Neotropical treefrogs composed of the genera *Aplastodiscus*, *Boana*, *Bokermannohyla*, *Hyloscirtus*, and *Myersiohyla*. The phylogenetic relationships of Cophomantini have been comprehensively reviewed in the literature, providing a suitable framework for the study of chromosome evolution. Employing different banding techniques, we studied the chromosomes of 25 species of *Boana* and 3 of *Hyloscirtus*; thus providing, for the first time, data for *Hyloscirtus* and for 15 species of *Boana*. Most species showed karyotypes with 2n = 2x = 24 chromosomes; some species of the *B*. *albopunctata* group have 2n = 2x = 22, and *H*. *alytolylax* has 2n = 2x = 20. Karyotypes are all bi-armed in most species presented, with the exception of *H*. *larinopygion* (FN = 46) and *H*. *alytolylax* (FN = 38), with karyotypes that have a single pair of small telocentric chromosomes. In most species of *Boana*, NORs are observed in a single pair of chromosomes, mostly in the small chromosomes, although in some species of the *B*. *albopunctata*, *B*. *pulchella*, and *B*. *semilineata* groups, this marker occurs on the larger pairs 8, 1, and 7, respectively. In *Hyloscirtus*, NOR position differs in the three studied species: *H*. *alytolylax* (4p), *H*. *palmeri* (4q), and *H*. *larinopygion* (1p). Heterochromatin is a variable marker that could provide valuable evidence, but it would be necesserary to understand the molecular composition of the C-bands that are observed in different species in order to test its putative homology. In *H*. *alytolylax*, a centromeric DAPI+ band was observed on one homologue of chromosome pair 2. The band was present in males but absent in females, providing evidence for an XX/XY sex determining system in this species. We review and discuss the importance of the different chromosome markers (NOR position, C-bands, and DAPI/CMA_3_ patterns) for their impact on the taxonomy and karyotype evolution in Cophomantini.

## Introduction

Hylidae is a monophyletic group of treefrogs with an almost worldwide distribution, and one of the most diverse families in the order Anura, with 969 recognized species [1]. Its members are grouped in the subfamilies Hylinae, Pelodryadinae, and Phyllomedusinae, the former composed of the tribes Cophomantini, Dendropsophini, Hylini, and Lophyohylini [2]. Large-scale phylogenetic studies are fairly congruent in terms of relationships of Hylinae (e.g. [3–7]).

Despite some discrepancies, the tribe Cophomantini has always been a well-supported monophyletic group, and a sister taxon of the remaining hylines. In addition, the intergeneric relationships of Cophomantini have been always recovered with the same topology in these analyses, with the genus *Myersiohyla* the earlier diverging lineage, and a sister taxon of a clade including *Hyloscirtus* (*Bokermannohyla* (*Aplastodiscus* + *Boana*)). Currently, Cophomantini includes 181 species in five genera: *Aplastodiscus* (15 spp.), *Boana* (92 spp.), *Bokermannohyla* (32 spp.), *Hyloscirtus* (36 spp.), and *Myersiohyla* (6 spp.).

Since the early revision by Faivovich et al. [2] the taxonomic knowledge and available phylogenetic hypotheses of genera within Cophomantini has greatly increased. A recent phylogeny by Berneck et al. [8] supports four monophyletic groups of *Aplastodiscus*: the *A*. *albofrenatus*, *A*. *albosignatus*, *A*. *perviridis*, and *A*. *sibilatus* species groups. The genus *Boana* (senior synonym of *Hypsiboas*, see [9]) contains seven species groups [2]: the *B*. *albopunctata*, *B*. *benitezi*, *B*. *faber*, *B*. *pulchella*, *B*. *pellucens*, *B*. *punctata*, and *B*. *semilineata* groups, for which 22 new species have been described subsequent to the analysis of Faivovich et al. [2] (see [1]). The relationships of these species groups have been addressed by disparate taxon sampling, with focus on the *B*. *pulchella* group [7,10–12], the *B*. *albopunctata* group [13], and the *B*. *semilineata* group [14]. The number of described species of *Bokermannohyla* increased to nine species since it was erected by Faivovich et al. [2]. In this genus, four species groups are recognized, *B*. *circumdata*, *B*. *claresignata*, *B*. *martinsi*, and *B*. *pseudopseudis*. Although the monophyly of the genus (and its species groups) is supported by some molecular studies, additional data is needed to confirm this [15].

The taxonomy and phylogeny of the genus *Hyloscirtus* (*sensu* [2]) were addressed in different contributions (i.e. [7,16–20]), and three species groups are recognized: *H*. *armatus*, *H*. *bogotensis*, and *H*. *larinopygion*. Rivera-Correa et al. [20] suggested the possibility of considering these three groups as different genera (or subgenera), but they also stressed the limited taxon sampling supporting the available phylogenetic hypotheses. Duellman et al. [7], however, proposed the creation of the genus *Colomascirtus* to include all species of the *H*. *armatus* and *H*. *larinopygion* groups, and restricted the genus *Hyloscirtus* to the taxa in the *H*. *bogotensis* group.

Finally, *Myersiohyla* was created by Faivovich et al. [2] to include species of the former *Hyla aromatica* group plus *H*. *kanaima*. The monophyly of this genus was rejected by Wiens et al. [4] and Pyron and Wiens [5], but a more inclusive subsequent analysis by Faivovich et al. [21] supported it as a natural group.

The data on hylid cytogenetics, with about 340 karyotyped species, makes it one of the most studied groups among Anura ([22], and references therein; [23–30]). Cytogenetic data are available for 230 spp. of Hylinae, followed by 80 spp. of Pelodryadinae, and 26 spp. of Phyllomedusinae, which account for 33, 38, and 43% of species in each clade respectively. Most pelodryadines and phyllomedusines share the basic number of x = 13 chromosomes, whereas in hylines karyotypes with x = 12 are frequently observed. Other basic chromosome numbers have been reported for Hylidae, albeit attributable to derived conditions. Supernumerary or B chromosomes described in seven hylines are another source of variation in chromosome number ([25], and references therein; [31–33]). Finally, sex chromosomes are a rare phemomenom in Hylidae, being restricted exclusively to Hylinae. They have been cytogenetically described in *Hyla femoralis*, *H*. *immaculata* (as *H*. *suweonensis*), *H*. *japonica*, *H*. *squirella* ([34,35], and references therein), and in *Pseudis tocantins* ([36], and references therein).

The phylogenetic position of Hylinae (i.e. sister of the clade composed of Pelodryadinae and Phyllomedusinae) suggests a possible synapomorphy of x = 12 for the subfamily [37], but as mentioned by Faivovich et al. [2] the distribution of this character in the most diverging lineages of the tribes of Hylinae requires study. Other information was then gathered from the genera *Phyllodytes* of Lophyohylini [38], and *Xenohyla*, *Scarthyla*, and *Sphaenorhynchus* of Dendropsophini [25]. Despite these new data, it is still necessary to study the chromosome number in the tribe Cophomantini, particularly in *Hyloscirtus* and *Myersiohyla*.

Recently, Duellman et al. [7] presented a new taxonomic arrangement for hylids, excluding from Hylidae the two former subfamilies Phyllomedusinae and Pelodryadinae, re-ranking them to the category of families. Most tribes of Hylinae were raised to subfamilies, some new genera erected, and some resurrected. The taxonomic changes proposed by these authors were not based on new evidence, nor on detailed discussions justifying most of them. As monophyly is not at question, we will follow the taxonomy employed by Faivovich et al. [2].

We have aimed in this study to increase our understanding of the cytogenetics of Cophomantini, the earlier diverging tribe of Hylinae. We provide for the first time chromosome data about *Hyloscirtus* (*sensu* Faivovich et al. [2]), describing the karyotypes of *H*. *alytolylax*, *H*. *palmeri*, and *H*. *larinopygion*. Additionally, we studied the chromosomes of 25 species of *Boana*, 15 of them for the first time. With these data and available information from the literature, we discuss the usefulness of some chromosome characters (NOR positions, C-bands and DAPI/CMA_3_ patterns) for the taxonomy and the interpretation of the chromosome evolution of Cophomantini, with special emphasis on *Boana*.

## Material and methods

We analyzed the karyotypes of 25 species of *Boana*, belonging to the following species groups: *B*. *albopunctata* (8 spp.), *B*. *faber* (1 sp.), *B*. *pellucens* (1 sp.), *B*. *pulchella* (10 spp.), *B*. *punctata* (2 spp.), and *B*. *semilineata* (3 spp.), and three species of *Hyloscirtus*, from the *H*. *bogotensis* and *H*. *larinopygion* groups. Specimens were treated with 0.1% colchicine, then euthanized with cutaneous application of 2% lidocaine under consent and approval of the the Ethical Committee in Animal Use (CEUA–permission number 027/2011, Instituto de Biociências, UNESP, Rio Claro, SP, Brazil), fixed in 4% formalin, and preserved in 70% ethanol. Specimen collections were approved by the following institutions: Argentina, Administración de Parques Nacionales (APN, PD-187/02), Ministerio de Ecología y Recursos Naturales Renovables (MEyRNR, 007/2009, 048/2013, 072/2014, 061/2015, 073/2016, and 035/2017), Programa de Recursos Naturales y Medio Ambiente (PRNyMA, 01/2016); Brazil, Instituto Chico Mendes de Conservação da Biodiversidade (ICMBio, Sistema de Autorização e Informação em Biodiversidade SISBIO, 30202–2, and 57098–1); Ecuador, Ministerio de Ambiente Ecuador (MAE, 005–12 IC-FAU-DNB/MA, and 003-17-IC-FAU-DNB/MA).

Chromosome preparations were obtained following Schmid et al. [32]. Chromosome relative length was measured using the software Micromeasure 3.3 [39] on mitotic metaphases stained with 10% buffered Giemsa solution, referring to the short and long arms as p and q repectively, and classified by using the centromeric index (CI) according to Green and Sessions [40]. We used the C-banding technique to detect constitutive heterochromatin [41], and the fluorochromes DAPI (4’,6-diamino-2-phenylindole) and CMA_3_ (chromomycin A_3_) to characterize the prevalence of different repetitive DNA sequences AT and CG, respectively [42]. The location of Nucleolar Organizer Regions (NORs) was described by performing silver staining [43] and fluorescence *in situ* hybridization (FISH) with a 18S rDNA labeled probe [44]. See supplementary on-line material (S1 File) for a list of collection sites, sex, and number of the specimens analyzed of each species, including museum voucher numbers and abbreviations.

We refer to the somatic chromosome number as 2n and to the basic chromosome number as x [45]. As usually occurs in other anurans, the establishment of chromosomal homologies in Cophomantini is problematic. In this sense, similar basic chromosome numbers in different taxa can be a consequence of different rearrangements and, therefore, non-homologous. Despite this constraint, with the available evidence we consider the basic chromosome number x as a character. This oversimplification allows us to perform the optimization of this character in a phylogenetic framework.

We optimized the basic chromosome number and the position of NORs of all the karyotypes described for Hylinae (see [22] for a review; [24–25,27–30,38], and references therein; see S1 Table for chromosome information in Cophomantini) on the phylogenetic hypothesis of Duellman et al. [7], as it is based on a reanalysis of most available hylid GenBank sequences, and is the latest and most inclusive phylogenetic hypothesis for the family. For the optimization of the basic number we considered the states of x = 9, 10, 11, 12, and 13 and for the chromosome location of NORs we considered NORs in pairs 1, 2, 4, 6, 8, 11, and 12. NORs in pair 4 in *Bokermannohyla alvarengai*, *Hyloscirtus alytolylax*, and *H*. *palmeri* are conspicuously different (see below), and therefore are considered as independent transformations. As suggested by Gruber et al. [46], the NOR bearing pair in *Aplastodiscus albosignatus* and *A*. *leucopygius* (pair 9) are likely homeologous to NOR bearing pairs in most species of cophomantines (pair 11), and thus are considered here as the same condition (i.e. as pair 11). Optimizations were done with TNT v1.1 [47], considering the character states of basic number and NOR position as unordered transformations.

## Results

We present cytogenetic data of 3 species of *Hyloscirtus* (*H*. *alytolylax*, *H*. *larinopygion* and *H*. *palmeri*) and 25 species of *Boana* (Table 1). The karyotypes of 3 species of *Hyloscirtus* and 12 taxa of *Boana* (*B*. *albonigra*, *B*. *almendarizae*, *B*. *caingua*, *B*. *calcarata*, *B*. *cipoensis*, *B*. cf. *alfaroi*, *B*. *heilprini*, *B*. *leucocheila*, *B*. *marianitae*, *B*. *pellucens*, *B*. *riojana*, and *B*. *stellae*) are studied for the first time.

**Table 1:** Studied species and the cytogenetic techniques applied for each one.

Species Group	Species	Locality	N	Differential Techniques
*H*. *bogotensis*	*H*. *alytolylax*	**Ecuador**. El Oro, Reserva Biológica Buenaventura.	2♀ 3♂	Ag-NORs, C-bands, DAPI/CMA_3_
	*H*. *palmeri*	**Ecuador**. Esmeraldas, Durango, Finca Sr. Antonio.	1♀	Ag-NORs, C-bands, DAPI/CMA_3_, rDNA
*H*. *larinopygion*	*H*. *larinopygion*	**Ecuador**. Carchi, Morán.	2♂	Ag-NORs, C-bands, DAPI/CMA_3_, rDNA
*B*. *albopunctata*	*B*. *almendarizae*	**Ecuador**. Tungurahua, Río Verde.	2♂	Ag-NORs, C-bands, DAPI/CMA_3_
	*B*. *calcarata*	**Ecuador**. Pastaza, Centro Etnoturístico Indichuris.	1♂	C-bands
	*B*. cf. *lanciformis*	**Brazil**. Amazonas, São Gabriel da Cachoeira.	1♂	Ag-NORs
	*B*. cf. *alfaroi*	**Brazil**. Pará, Dique, Carajas.	1♂ 1u	rDNA
	*B*. *heilprini*	Pet trade.	1♀ 1♂	DAPI/CMA_3_
	*B*. *leucocheila*	**Brazil**. Pará, Jurutí, Acampamento Barroso; Mutúm.	3♂	Ag-NORs,DAPI/CMA_3_, rDNA
	*B*. *multifasciata*	**Brazil**. Pará, Igarapé-Miri, Vila Maiauata; Curuçá, Ilha Pedras Grandes.	3♂ 1u	Ag-NORs, C-bands, DAPI/CMA_3_, rDNA
	*B*. *raniceps*	**Argentina**. Misiones, Capital, Posadas. Chaco, San Fernando; General Güemes, near Misión Nueva Pompeya. Salta, Orán, near Pichanal. Corrientes: San Miguel, Estero Carambola; **Brazil**: Piauí, PNSC-PI; Nazaré; Pará, Soure.	1♀ 14♂ 1j 2u	Ag-NORs, C-bands, DAPI/CMA_3_, rDNA
*B*. *faber*	*B*. *faber*	**Argentina**. Misiones, Cainguás, Aristóbulo del Valle, Balneario Arroyo Cuña Pirú; San Pedro, Tobuna.	3♂	Ag-NORs, C-bands, DAPI/CMA_3_, rDNA
*B*. *pellucens*	*B*. *pellucens*	**Ecuador**. Esmeraldas, Durango, Finca Sr. Antonio; Muisne—Cabo San Francisco, Laguna del Diablo.	2♂	Ag-NORs, C-bands, DAPI/CMA_3_, rDNA
*B*. *pulchella*	*B*. *albonigra*	**Argentina**. Jujuy, Santa Catalina, Santa Catalina; El Queñoal.	1♀ 2♂	Ag-NORs, C-bands, DAPI/CMA_3_
	*B*. *caingua*	**Argentina**. Misiones, Capital, Posadas; Candelaria, Candelaria.	1♀ 12♂	Ag-NORs, C-bands, DAPI/CMA_3_
	*B*. *cipoensis*	**Brazil**. Minas Gerais, Serra do Cipó; Diamantina.	4♂	Ag-NORs, C-bands, DAPI/CMA_3_, rDNA
	*B*. *cordobae*	**Argentina**. Córdoba, Colón, Quebrada del Cóndor. San Luis, Coronel Pringles, near Inti Huasi.	2♀ 9♂	Ag-NORs, C-bands, DAPI/CMA_3_
	*B*. *curupi*	**Argentina**. Misiones, Gral. Manuel Belgrano, Península Andresito.	6♂	Ag-NORs, C-bands, DAPI/CMA_3_
	*B*. *bischoffi*	**Brazil**. São Paulo, Apiaí, Morro do Ouro.	1♂	Ag-NORs, C-bands
	*B*. *marianitae*	**Argentina**. Salta, Santa Victoria, Parque Nacional Baritú.	2♂	Ag-NORs, C-bands, DAPI/CMA_3_
	*B*. *pulchella*	**Argentina**. Buenos Aires, Tandil; La Plata. Misiones, Candelaria, Santa Ana; Profundidad; Cerro Corá; Campo San Juan; Apóstoles. Entre Ríos, near Brazo Largo. Corrientes, Santo Tomé, Gobernador Virasoro; Monte Caseros; Curuzú Cuatiá, Perugorría. San Luis, El Volcán, La Hoya. **Uruguay**. Rocha, Laguna Negra; Flores, near Trinidad; San José, Libertad.	3♀ 34♂ 1j	Ag-NORs, C-bands, DAPI/CMA_3_
	*B*. *riojana*	**Argentina**. La Rioja, Chilecito, near El Siciliano; Famatina, Cuesta la Aguadita. Salta, Rosario de Lerma, Encón Grande. Catamarca, El Alto, Sierras de Ancasti; Guayamba, Río Guayamba. Tucumán, Tafí del Valle, Quebrada Zanja de los Cardones; Camping Los Sauzales.	6♀ 8♂	Ag-NORs, C-bands, DAPI/CMA_3_, rDNA
	*B*. *stellae*	**Argentina**. Misiones, Cainguás, Balneario Arroyo Cuña Pirú. Parque Provincial Salto Encantado.	1♀ 8♂	Ag-NORs, C-bands, DAPI/CMA_3_, rDNA
*B*. *punctata*	*B*. *cinerascens*	**Ecuador**. Tungurahua, Rio Negro. **Brazil**. Pará, Santa Barbara do Pará, Gunma; Curuçá, Nazaré de Mocajuba; Boa Vista do Muriá. Amazonas, São Gabriel da Cachoeira.	7♂ 1u	Ag-NORs, C-bands, DAPI/CMA_3_, rDNA
	*B*. *punctata*	**Brazil**. Pará, Marabá, Marabá; Maranhão, Municipality of Porto Franco.	2♀	Ag-NORs, C-bands, DAPI/CMA_3_, rDNA
*B*. *semilineata*	*B*. *boans*	**Brazil**. Pará, Melgaço, Caxiuana; Carajás; Marabá, Piçarreira.	2♂ 1u	Ag-NORs, C-bands, DAPI/CMA_3_, rDNA
	*B*. cf. *semilineata*	**Brazil**. Pará: Juruti, Mutúm; Marabá.	1♂ 1u	Ag-NORs, C-bands, DAPI/CMA_3_, rDNA
	*B*. *wavrini*	**Brazil**. Pará, Melgaço, Caxiuana.	1♂ 2u	Ag-NORs, C-bands, DAPI/CMA_3_, rDNA

N = Number of specimens analyzed. j = juvenile, u = undetermined.

The karyotypes of *Boana multifasciata*, *B*. cf. *lanciformis*, and *B*. cf. *semilineata* present remarkable differences to those described for *B*. *multifasciata*, *B*. *lanciformis* [48], and *B*. *semilineata* [49]. We additionally redescribe the karyotypes of eight species with new techniques: Ag-NORs (*B*. *cordobae*, *B*. *pulchella*, and *B*. *punctata*), C- bands (*B*. *punctata*), DAPI/CMA_3_ patterns (*B*. *cinerascens*, *B*. *cordobae*, *B*. *curupi*, *B*. *multifasciata*, *B*. *pulchella*, *B*. *punctata*, *B*. *raniceps*, and *B*. *wavrini*), and FISH by using 18S rDNA probes (*B*. *boans*, *B*. *cinerascens*, *B*. *multifasciata*, *B*. *raniceps*, *B*. *punctata*, and *B*. *wavrini*).

**Fig 1:**
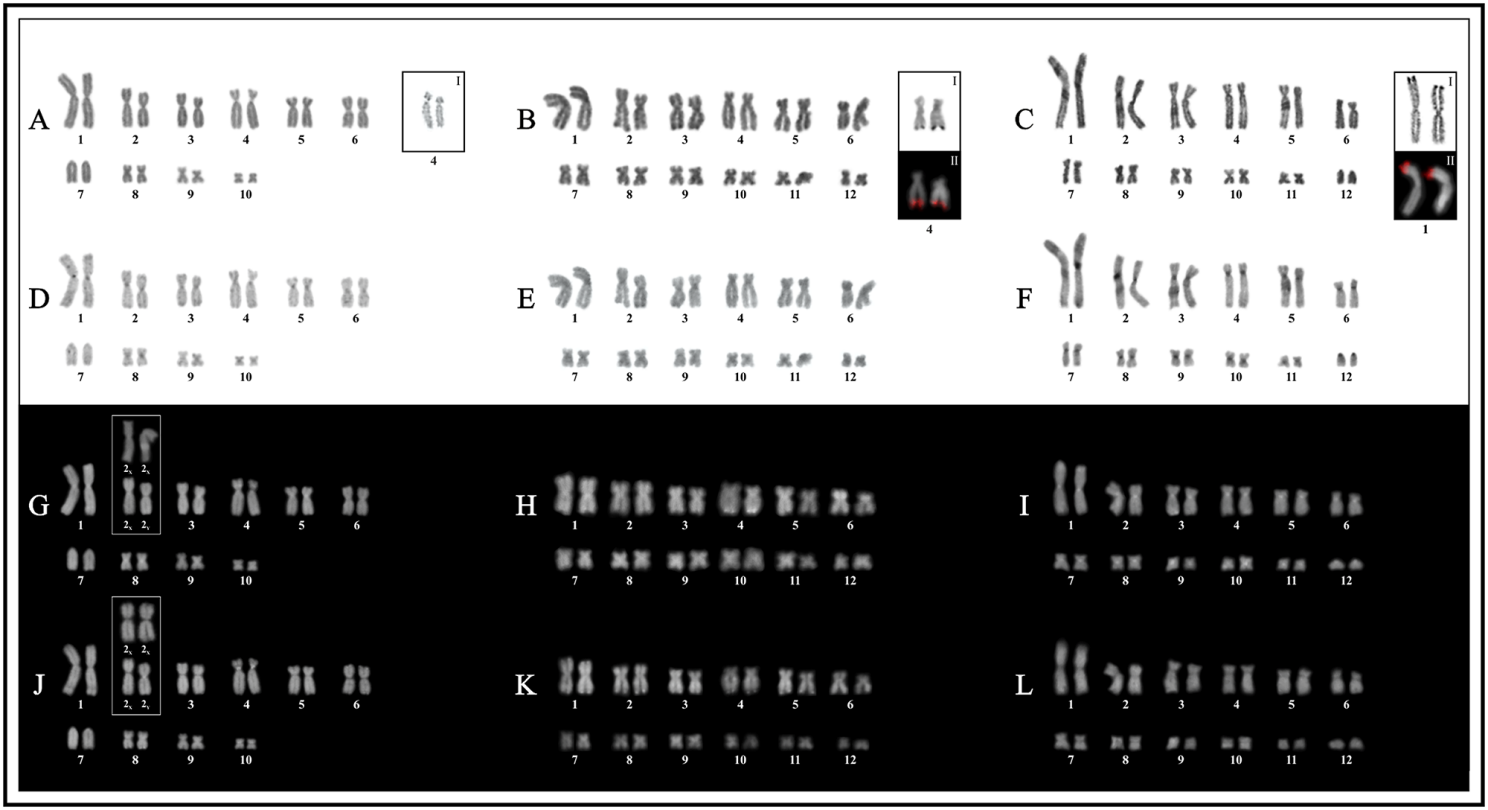
Karyotypes of three species of *Hyloscirtus*. *Hyloscirtus alytolylax* (left), *H*. *palmeri* (center), and *H*. *larinopygion* (right). **A, B.** Giemsa staining. **C, D.** C-bands. **E, F.** CMA_3_. **G, H.** DAPI. Squares show NOR-bearing chromosome pairs as stained by the silver impregnation technique (**I**), and with FISH using a 18S DNA probe (**II**).

**Fig 2:**
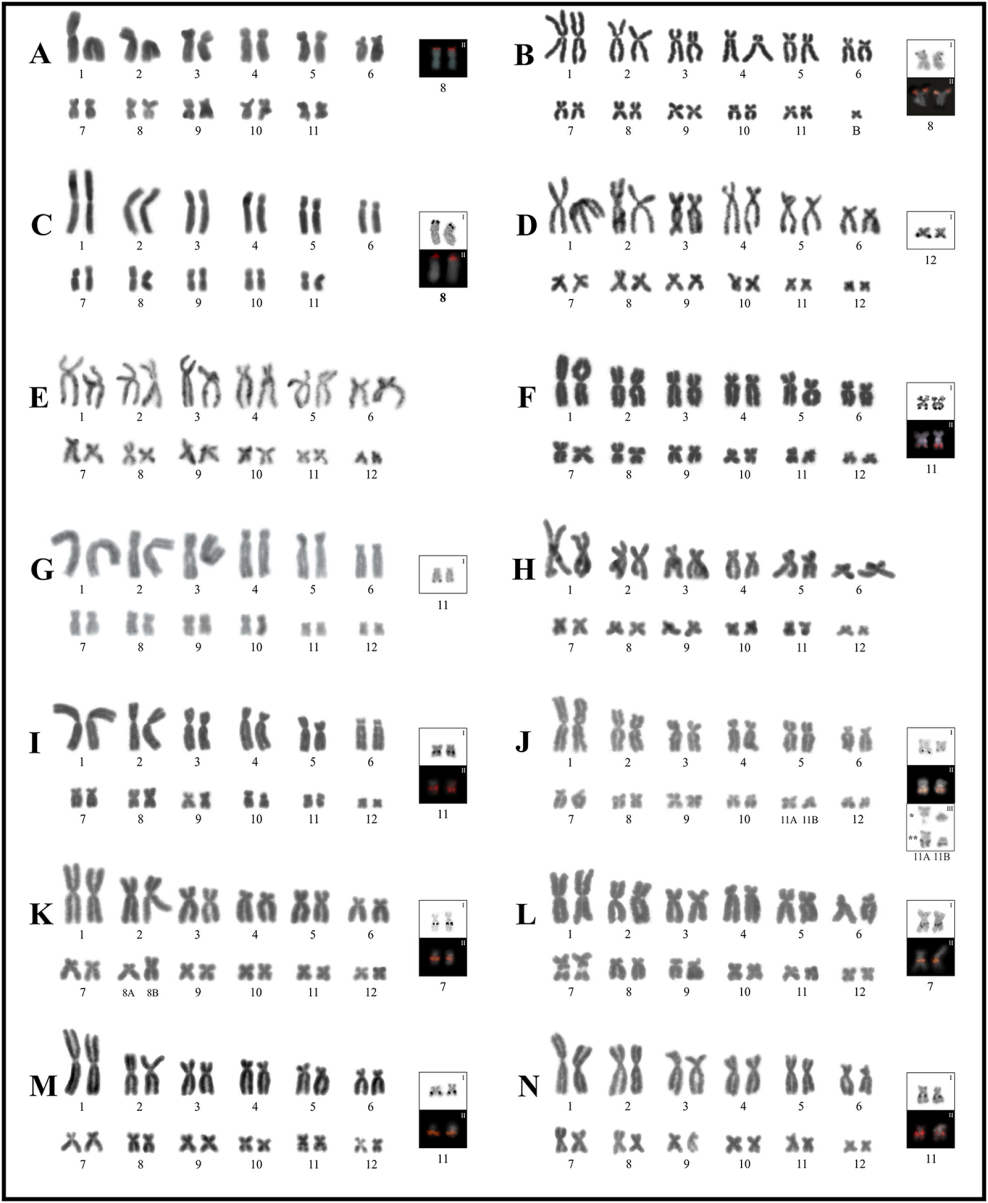
Giemsa stained karyotypes of the *Boana albopunctata*, *B*. *punctata*, *B*. *semilineata*, and *B*. *pellucens* groups. **A.** *Boana* cf. *alfaroi*. **B.** *B*. *leucocheila*. **C.** *B*. *multifasciata*. **D.** *B*. *almendarizae*. **E.** *B*. *heilprini*. **F**. *B*. *raniceps*. **G.** *B*. cf. *lanciformis*. **H.** *B*. *calcarata*. **I.** *B*. *cinerascens*. **J.** *B*. *punctata*. **K.** *B*. *boans*. **L**. *B*. cf. *semilineata*. **M.** *B*. *wavrini*. **N.** *B*. *pellucens*. Squares show NOR-bearing chromosome pairs as stained by the silver impregnation technique (**I**), and with FISH using a 18S DNA probe (**II**). Sequential Giemsa (*) and Ag-NORs (**) staining on chromosomes 11A and 11B from another female of *B*. *punctata* (CFBH 39626) are shown in (**III)**.

**Fig 3:**
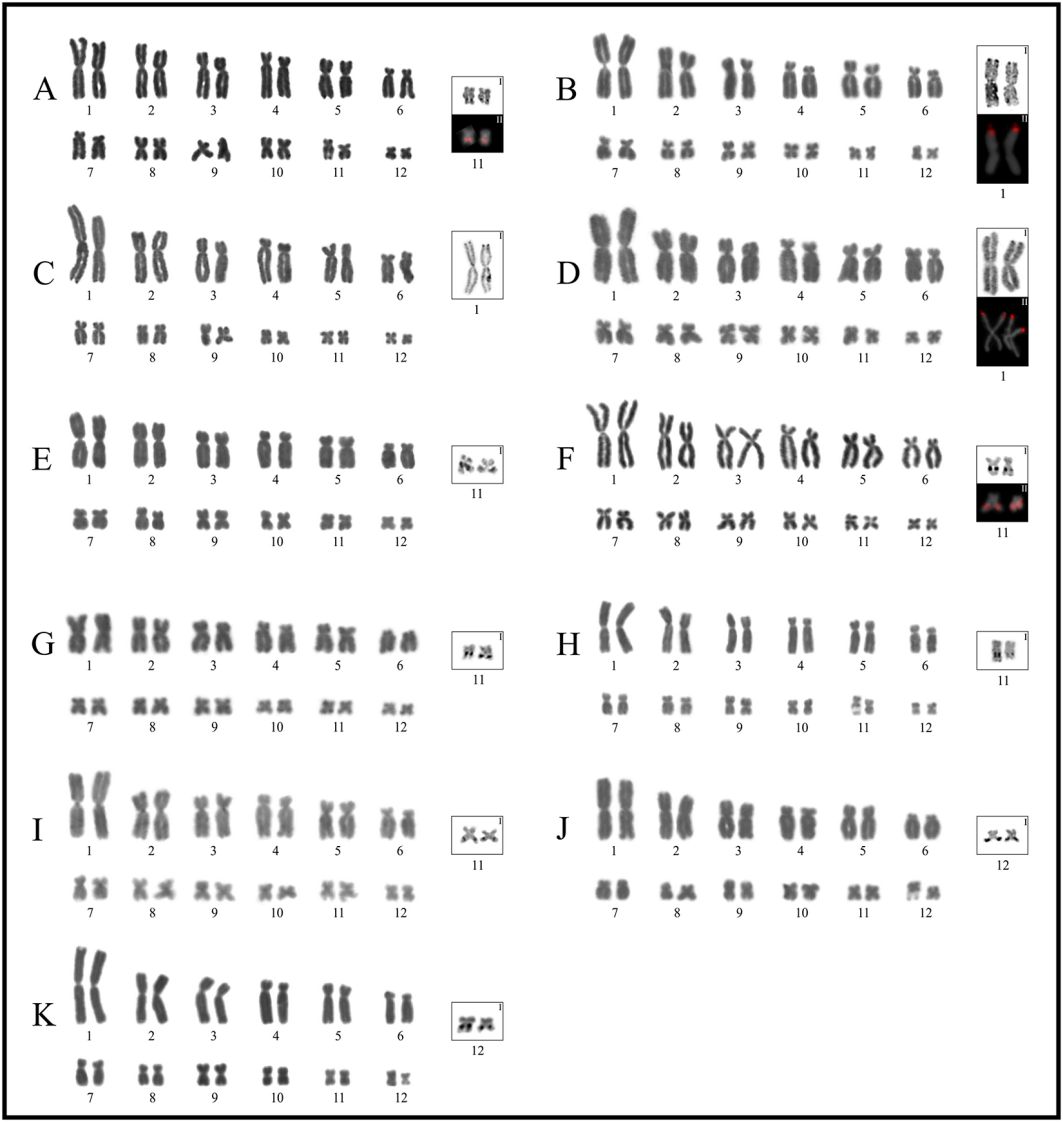
Giemsa stained karyotypes of the *Boana faber* and *B*. *pulchella* groups. **A.** *Boana faber*; **B.** *B*. *cipoensis*; **C.** *B*. *curupi*; **D.** *B*. *stellae*; **E.** *B*. *albonigra*; **F.** *B*. *riojana*; **G.** *B*. *marianitae*; **H.** *B*. *bischoffi*; **I.** *B*. *cordobae*; **J.** *B*. *pulchella*; **K.** *B*. *caingua*. Squares show NOR-bearing chromosome pairs characterized by silver impregnation technique (**I**), and by FISH with a 18S DNA probe (**II**).

**Fig 4:**
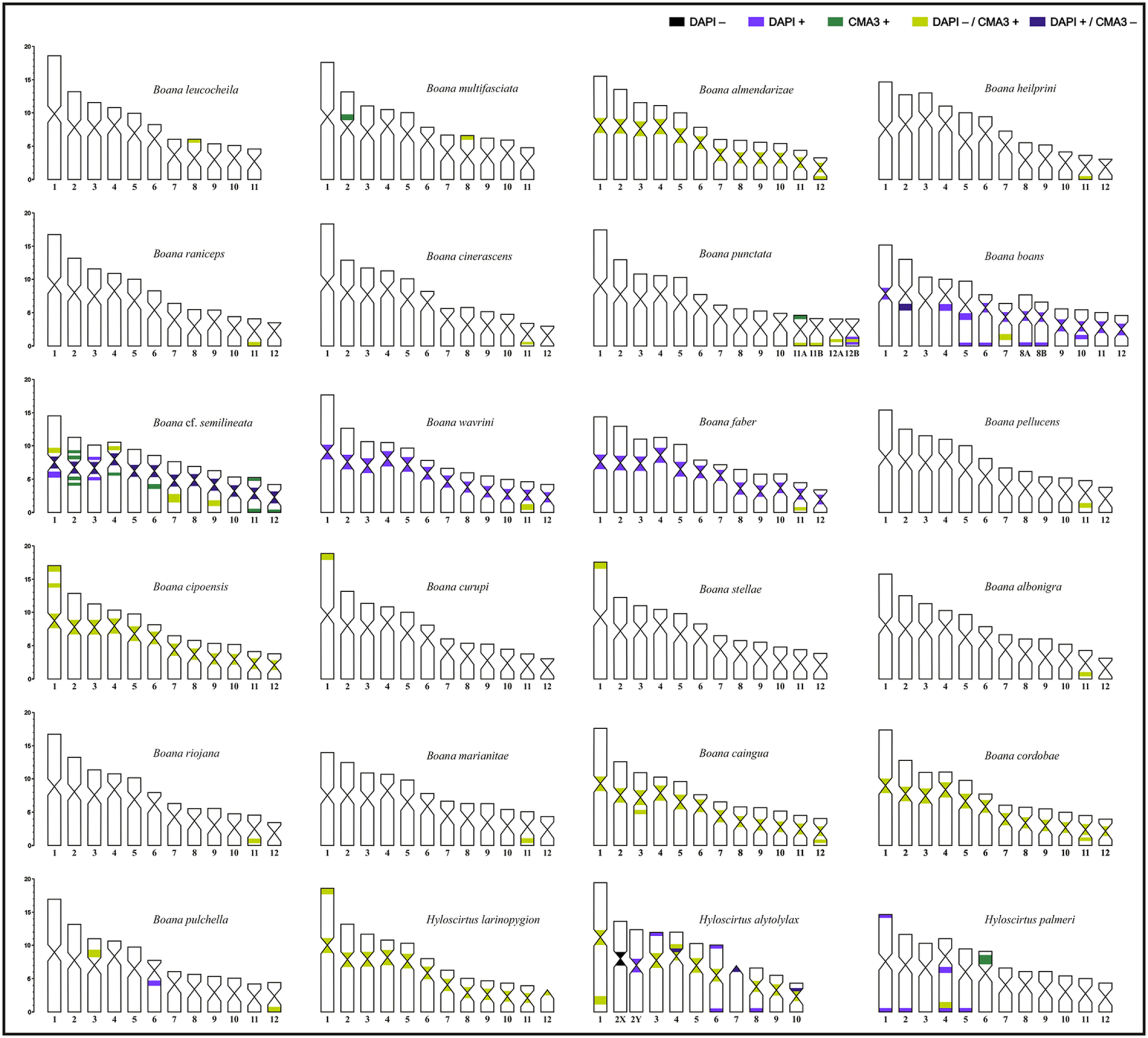
Idiograms with DAPI-CMA_3_ bands present in the species of *Boana* and *Hyloscirtus* studied in this paper. Scale indicates percentage relative size of chromosome pairs.

A karyotype with 2n = 2x = 24 chromosomes was observed in most species, except for some members of the *Boana albopunctata* group, that have 2n = 2x = 22, and for *Hyloscirtus alytolylax*, that have 2n = 2x = 20. Almost all species presented karyotypes with bi-armed chromosomes, with the exception of *H*. *alytolylax* and *H*. *larinopygion*, whose karyotypes have a single pair of small telocentric chromosomes, but of different sizes (Figs 1, 2, 3 and 4). The chromosome morphology of each species is summarized in S2 Table. In all karyotypes a single pair of chromosomes bearing Ag-NOR sites was observed, generally associated to secondary constrictions, in all cases co-locating with DAPI-/CMA_3_+ staining and with the 18S rDNA hybridization signals. In Table 1 we summarize the studied species and the cytogenetic techniques applied for each species.

**Fig 5:**
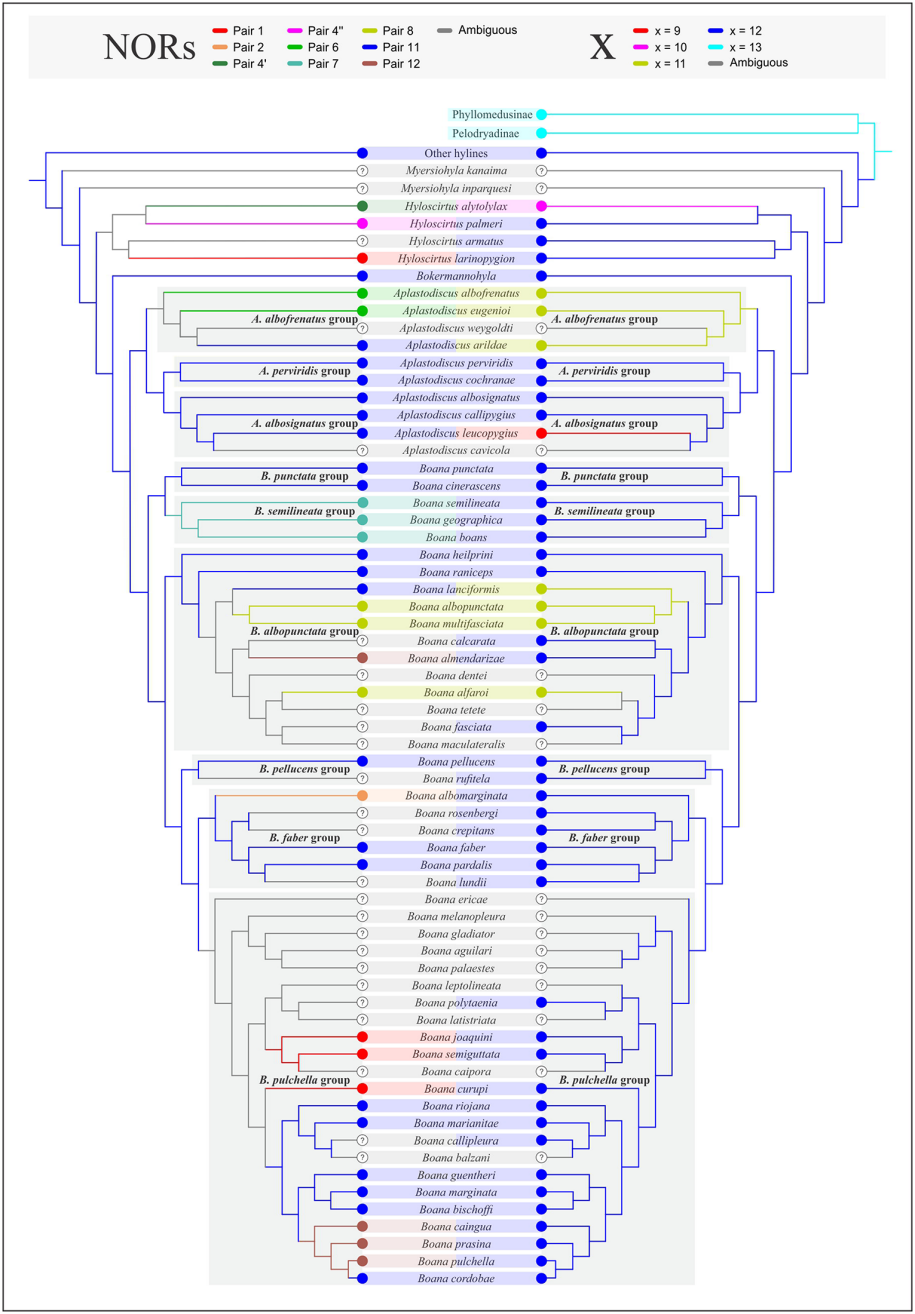
Optimization of the basic number (right) and the position of NORs (left) in Cophomantini on a condensed phylogenetic hypothesis resulting from the analyses of Faivovich et al. [21] and Duellman et al. [7].

The optimization of basic chromosome number and NORs are shown in Fig 5 and S1 Fig. A basic number of x = 12 was recovered as a synapomorphy of Hylinae, while other numbers represent synapomorphies of less inclusive clades in *Aplastodiscus* (x = 10 and 11) and *Boana* (x = 11). On the other hand, NORs in pair 11 optimized as a synapomorphy for Hylinae, with NORs in pairs 7 and 12 being synapomorphies for less inclusive clades in the *Boana semilineata* and *B*. *pulchella* groups, respectively.

### Hyloscirtus

In *Hyloscirtus alytolylax* pairs 1–2, 6, 8–10 were metacentric, pairs 3–5 submetacentric, and pair 7 was telocentric. In the other two species, pairs 1, 2, 9–11 were metacentric, pairs 3, 5, and 6 submetacentric, and pair 4 was subtelocentric. In *H*. *palmeri* pair 7 was metacentric, pair 8 submetacentric, and pair 12 metacentric, while in *H*. *larinopygion*, these pairs were submetacentric, metacentric, and telocentric, respectively.

NORs were detected in the terminal regions of pair 1p in *Hyloscirtus larinopygion*, pair 4p in *H*. *alytolylax*, and pair 4q in *H*. *palmeri* (Fig 1A–1C). The karyotypes of these species showed heterochromatic C-bands in most centromeres, interstitially in 1q and 4p in *H*. *alytolylax*, and in 6p in *H*. *palmeri* (Fig 1D–1F). GC-rich heterochromatin (DAPI-/CMA_3_+) was present interstitially in pair 1, and in the centromeres of pairs 1, 3–5, 6, 8, 9 and 10 of *H*. *alytolylax*, in almost the complete arm of 6p in *H*. *palmeri*, and in all centromeres in *H*. *larinopygion*. Heterochromatic DAPI+ bands were observed in *H*. *alytolylax* in proximal position on pairs 4p and 10p, terminally on pairs 3p, 6p6q and 8q, and in the centromeric region of pair 7, and in *H*. *palmeri*, in a terminal position on pairs 1p1q, 2q, 4q, and 5q, and interstitially in pair 4q (Figs 1G–1L and 4).

An additional centromeric DAPI+ band was present in one of the homologues of chromosome pair 2 in males of *Hyloscirtus alytolylax* (2Y). The presence of this heteromorphic band was present in males and absent in females, where in the latter, centromeres of both chromosomes of pair 2 showed a similar DAPI- pattern (2X). Thirty DAPI-stained metaphases were measured (15 of males and 15 of females), showing that chromosome 2Y was slightly smaller than chromosome 2X, although both elements are similar in shape (S2 Table).

### The *Boana albopunctata* group

Two different chromosome numbers were identified in this group. *Boana* cf. *alfaroi*, *B*. *leucocheila*, and *B*. *multifasciata* shared 2n = 2x = 22 chromosomes (Fig 2A–2C), while *B*. *almendarizae*, *B*. *calcarata*, *B*. cf. *lanciformis*, *B*. *heilprini*, and *B*. *raniceps* presented 2n = 2x = 24 (Fig 2D–2H).

In *Boana* cf. *alfaroi*, *B*. *leucocheila*, and *B*. *multifasciata* pairs 1, 2, 8, 9 and 11 were metacentric, and pairs 3, 5, and 10 submetacentric. Chromosome pair 7 was submetacentric in *B*. cf. *alfaroi* and *B*. *multifasciata*, and metacentric in *B*. *leucocheila*. In *B*. *multifasciata* pairs 4 and 6 were subtelocentric, while in the two other species these pairs were submetacentric.

In the five species with 2n = 24, pairs 1, 2, and 8 were metacentric, and pair 3 submetacentric. Pair 4 was subtelocentric in *B*. *calcarata*, *B*. cf. *lanciformis* and *B*. *raniceps*, and submetacentric in *B*. *almendarizae* and *B*. *heilprini*. In *B*. *almendarizae*, *B*. *calcarata* and *B*. *raniceps* pairs 5 and 6 were submetacentric, whereas these pairs were respectively metacentric and submetacentric in *B*. *heilprini* and, submetacentric and subtelocentric in *B*. cf. *lanciformis*. In *B*. *calcarata* and *B*. *raniceps*, pair 7 was metacentric, and in the other species, *B*. *almendarizae*, *B*. *heilprini* and *B*. cf. *lanciformis* were submetacentric. Moreover, pair 9 was metacentric in *B*. *calcarata* and *B*. cf. *lanciformis*, but submetacentric in *B*. *almendarizae*, *B*. *heilprini*, and *B*. *raniceps*. Pairs 10 and 11 were metacentric in *B*. *almendarizae*, *B*. *calcarata*, and *B*. *heilprini* and *B*. *raniceps*, and submetacentric in *B*. cf. *lanciformis*, the latter being a distinctive feature of this species. Finally, the smallest pair 12 was metacentric in *B*. *almendarizae*, *B*. *calcarata* and *B*. cf. *lanciformis*, and submetacentric in *B*. *heilprini* and *B*. *raniceps*.

NORs were located terminally on pair 8p in *B*. cf. *alfaroi*, *B*. *leucocheila*, and *B*. *multifasciata*, on pair 11q in *B*. cf. *lanciformis* and *B*. *raniceps*, and on pair 12 in *B*. *almendarizae* (Fig 2A–2H).

**Fig 6:**
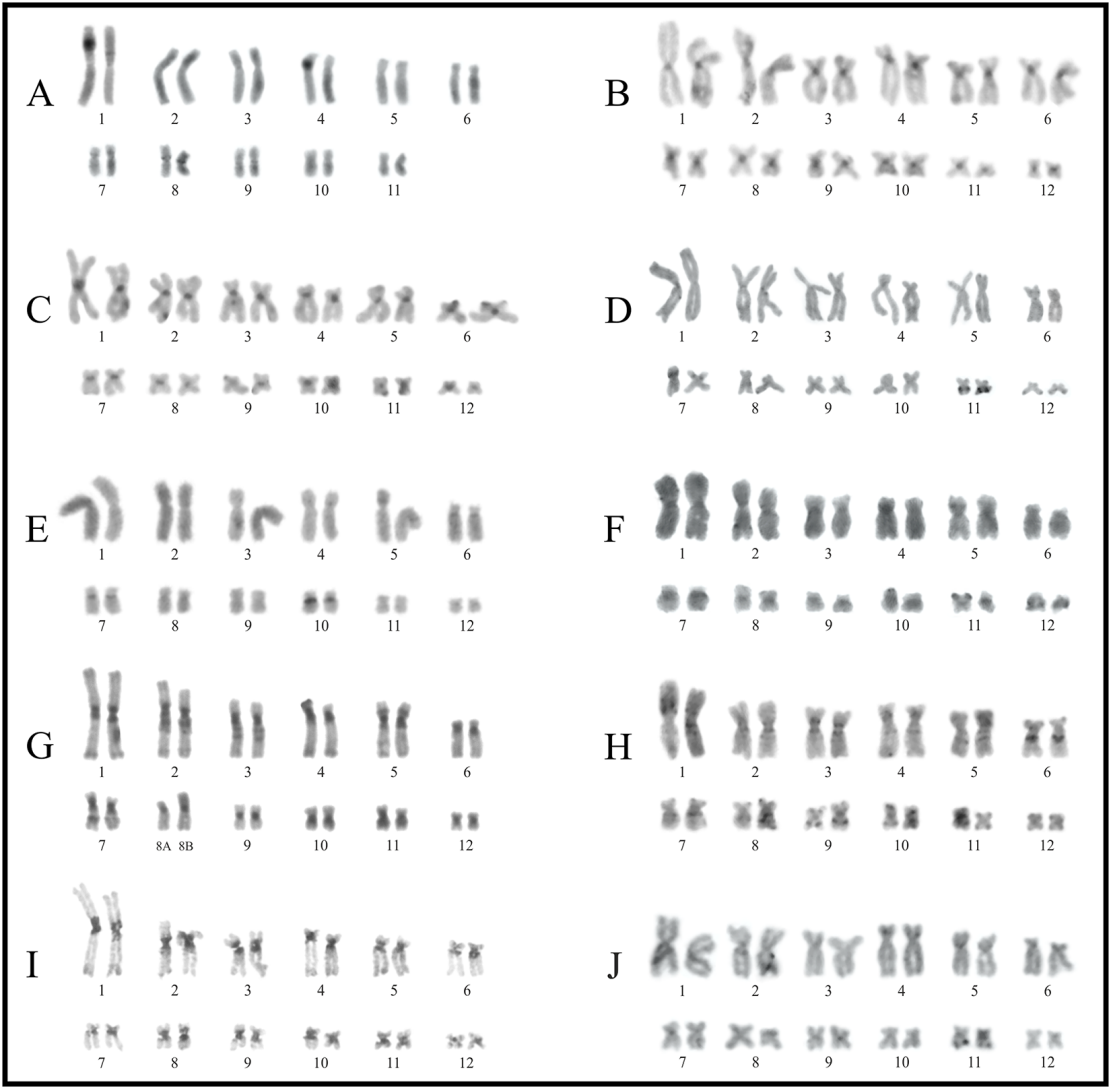
C banding pattern of the *Boana albopunctata*, *B*. *punctata*, *B*. *semilineata*, and *B*. *pellunces* groups. **A.** *B*. *multifasciata*. **B.** *B*. *almendarizae*. **C**. *B*. *calcarata*. **D.** *B*. *raniceps*. **E.** *B*. *cinerascens*. **F.** *B*. *punctata*. **G.** *B*.*boans*. **H**. *B*. cf. *semilineata*. **I.** *B*. *wavrini*. **J.** *B*. *pellucens*.

In *Boana multifasciata* C-bands occured only on pairs 1, 7, 8, and 9; interstitially on 1p and 7p7q, and pericentromerically on 8p and 9q (Fig 6A). *Boana almendarizae* and *B*. *calcarata* showed a similar heterochromatic pattern restricted exclusively to all centromeres (Fig 6B and 6C), whereas in *B*. *raniceps* C+ heterochromatin was pericentromeric and interstitial on pairs 7p and 11q, respectively (Fig 6D).

No DAPI+ bands were observed in any of the species of this group. Moreover, in *Boana almendarizae* the heterochromatin of all centromeres were DAPI-/CMA_3_+ (Fig 4, S2 Fig). Due to the quality of preparations, we could not establish the position of the NORs in *B*. *heilprini*, although we observed conspicuous terminal DAPI-/CMA_3_+ signals on pair 11 (S2 Fig). Given that pattern of well-delimitated fluorescent signals (DAPI-/CMA_3_+) corresponds to NOR sites for most vertebrates (see [32] for a review); it is plausible that NORs are also located terminally on pair 11 in *B*. *heilprini*.

Additionally, two specimens of *Boana leucocheila* exhibited one mitotically stable, small sized (3.11% of the haploid set) B chromosome with a metacentric morphology (CI: 0.46). This chromosome showed no hybridization with rDNA.

### The *Boana punctata* group

In *Boana cinerascens* and *B*. *punctata*, pairs 1, 2, 8 and 9 were metacentric, pairs 3, 5, 7 and 10 submetacentric, and pair 6 subtelocentric. In *B*. *cinerascens*, pair 4 is subtelocentric and pair 12 metacentric, whereas in *B*. *punctata* these pairs are submetacentric. Two females of *Boana punctata* (PS 855, CFBH 39626) had the chromosomes of pair 11 morphologically different, one metacentric (11A) and the other submetacentric (11B), this pair was metacentric in *B*. *cinerascens*.

In *Boana cinerascens* NORs were located interstitially on pair 11q (Fig 2I). The karyotype of *B*. *cinerascens* only showed pericentromeric heterochromatin on pairs 5p and 10q (Fig 6E).

In one sample of *Boana punctata* (CFBH 39626), Ag-NORs were heteromorphic in size; while in the other (PS 855), no differences were observed for either Ag-NORs or rDNA hybridization signals detected by FISH. Both chromosomes, 11A and 11B, were different in respect to C-bands and DAPI/CMA_3_ pattern, with 11A having an additional terminal C-band (DAPI-/CMA_3_+) on 11p. Homologues of pair 12 have interstitial heterochromatin DAPI-/CMA_3_+, but one of them showed additional interstitial heterochromatin DAPI+ (Figs 2J, 4 and 6F, S2 Fig).

### The *Boana semilineata* group

In *Boana boans*, *B*. cf. *semilineata* and *B*. *wavrini* pairs 1, 2, 10–12 were metacentric, pairs 3, 5–8 submetacentric, and pair 4 was subtelocentric. Although pair 5 was submetacentric in the three species, in *B*. *boans* and *B*. cf. *semilineata* it had a remarkable submetacentric morphology (CI: 0.35 and 0.34, respectively); in *B*. *wavrini* this pair was almost subtelocentric (CI: 0.25). Pair 9 was metacentric in *B*. *boans* and *B*. *wavrini*, and submetacentric in *B*. cf. *semilineata*.

Karyotypes of the three species showed interstitial NORs, associated with heterochromatin in *Boana boans* and *B*. cf. *semilineata*. In *B*. *wavrini* NORs were on pair 11q, whereas in *B*. *boans* and *B*. cf. *semilineata* on 7q (Fig 2K–2M). The three species showed a conspicuous C-banding pattern on the centromeric and pericentromeric regions of most chromosomes (Fig 6G–6I). Additional interstitial C-bands were evident, in *B*. *boans* on pairs 2q, 3q, 5p, and 7q, and in *B*. cf. *semilineata* on pairs 1–9. In *B*. cf. *semilineata*, smaller pairs (10–12) have additional heterochromatin restricted to a terminal location (Fig 6I).

Fluorochromes DAPI and CMA_3_ produced bright signals in most chromosomes of the three species, but with distinctive patterns in each one (Fig 4, S2 Fig). In *Boana boans* AT-rich heterochromatic bands (DAPI+) are present on centromeric and pericentromeric positions of pairs 1, 2 (also CMA_3_-), 4–12, and terminal on pairs 5, 6 and 8. In *B*. *wavrini* DAPI+ heterochomatin is present in almost all centromeres. In both species, CMA_3_ signals are restricted to NORs regions (DAPI-). *Boana* cf. *semilineata* presented the bright DAPI+ heterochromatin pericentromeric on one arm of pair 1, on both arms of pair 3, and on the centromeres of all chromosomes that are CMA_3_-. On the other hand, CMA_3_+ signals are interstitial on pairs 2, 4, and 6, and distal on pairs 11 and 12. DAPI-/CMA_3_+ signals are evident on interstitial positions of pairs 1, 4, 7, and 9.

One of the three studied males of *Boana boans* (PS 785) showed a heteromorphism between homologues of pair 8 (Figs 2K and 6G), which was composed by a submetacentric chromosome (8A, CI: 0.34; commonly observed in the other karyotypes of the species), and a metacentric one (8B, CI: 0.41). No differences were detected in regard to the banding patterns of these elements.

### The *Boana faber* and *B*. *pellucens* groups

**Fig 7:**
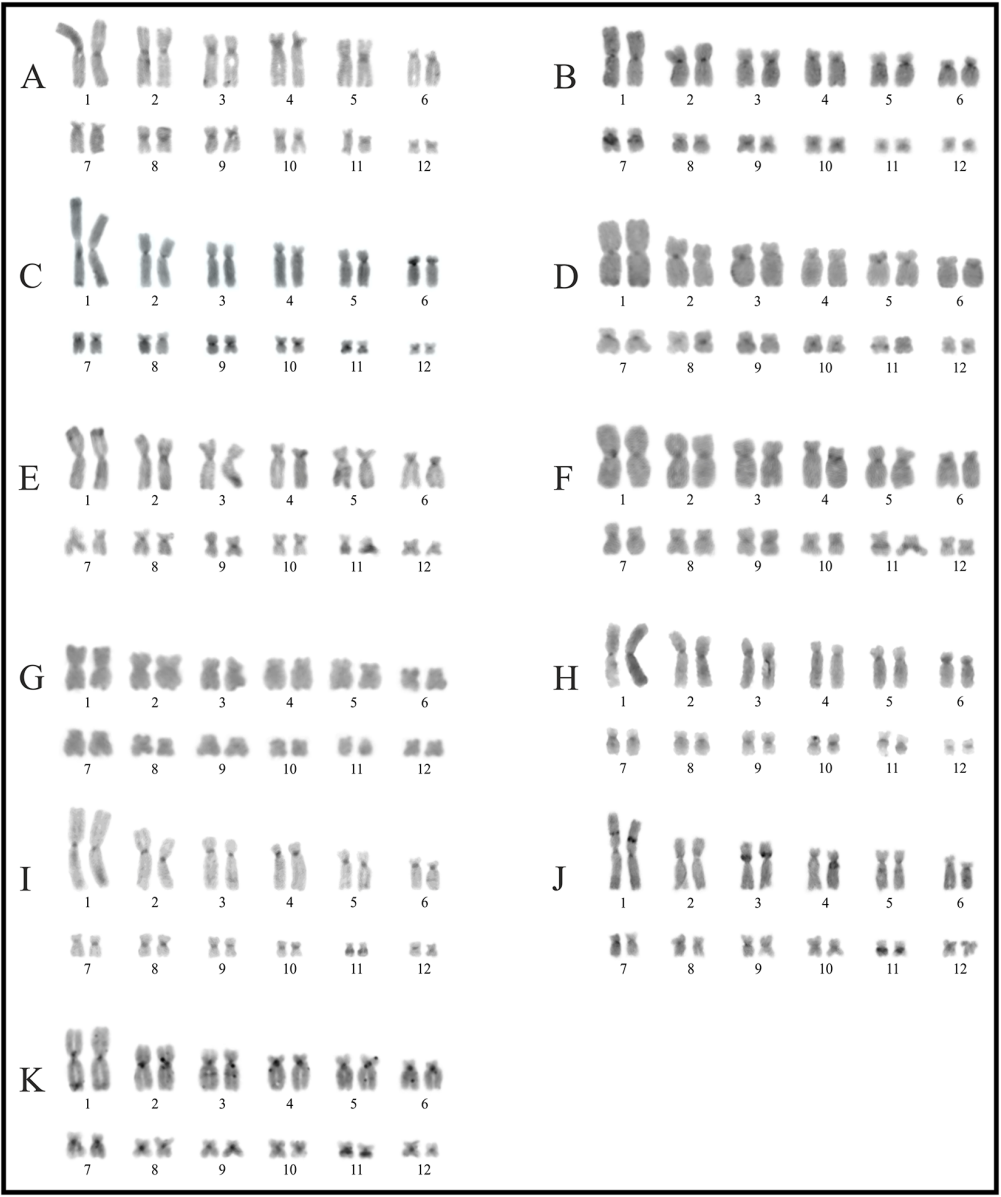
C banding pattern in karyotypes of the *Boana faber* and *B*. *pulchella* groups. **A.** *B*. *faber*. **B.** *B*. *cipoensis*. **C.** *B*. *curupi*. **D.** *B*. *stellae*. **E.** *B*. *albonigra*. **F.** *B*. *riojana*. **G.** *B*. *marianitae*. **H.** *B*. *bischoffi*. **I.** *B*. *cordobae*. **J.** *B*. *pulchella*. **K.** *B*. *caingua*.

In *Boana pellucens* pairs 1, 2, 7, 8, 10 and 12 were metacentric, and pairs 3–6, 9 and 11 submetacentric. NORs were interstitial on pair 11q (Fig 2N). This species showed mostly a centromeric C-banding pattern, with pericentromeric heterochromatin on pairs 2q, 4p, and 5p, but distal on pair 11q (Fig 6J). In *B*. *faber* chromosome pairs 1, 2, 8, 11 and 12 were metacentric, pairs 3, 5, 9 and 10 submetacentric and, pairs 4, 6, and 7 subtelocentric. In this species NORs were terminally located on pair 11q (Fig 3A), constitutive hererochromatin was detected on the centromeres of all chromosomes (Fig 7A), and all chromosomes had centromeric DAPI+ signals (Fig 4, S2 Fig).

### The *Boana pulchella* group

In all species, pairs 1, 2, 9–11 and 12 were metacentric, pairs 3, 5 and 7 submetacentric and, pairs 4 and 6 subtelocentric. Subtle differences were observed in the morphology of the pair 8. This pair was metancentric in *Boana caingua*, *B*. *cordobae* and *B*. *pulchella*, and submetacentric in *B*. *albonigra*, *B*. *cipoensis*, *B*. *curupi*, *B*. *bischoffi*, *B*. *marianitae*, *B*. *riojana*, and *B*. *stellae*.

In this group NORs were found in different locations: (A) terminally on pair 1p in *Boana cipoensis*, *B*. *curupi*, and *B*. *stellae* (Fig 3B–3D); (B) on pair 11q in *B*. *albonigra*, *B*. *bischoffi*, *B*. *marianitae*, and *B*. *riojana* (interstitial), and *B*. *cordobae* (terminal) (Fig 3E–3I); (C) on pair 12 in *B*. *caingua* (interstitial) and *B*. *pulchella* (terminal) (Fig 3J and 3K).

In most karyotypes, C-bands were observed on the centromeres, although additional bands were evident in some species (Fig 7B–7K). Interstitial heterochromatin occurs on pairs 3q and 11q in *Boana caingua*, and on pair 11q in *B*. *albonigra*, *B*. *cordobae*, *B*. *curupi*, *B*. *puchella*, *B*. *riojana*, and *B*. *stellae*. Pericentromeric C-bands were detected on pair 3p in *B*. *pulchella*, pair 6p in *B*. *curupi*, and pair 7q in *B*. *cipoensis*. Terminal C-bands were present on chromosome pair 1p in *B*. *albonigra*, *B*. *caingua*, *B*. *curupi*, and *B*. *stellae*, and pair 12q in *B*. *pulchella* and *B*. *caingua*, but the detection of these terminal bands varied among metaphases. In *B*. *pulchella*, we detected a conspicuous interstitial heterochromatic band on pair 1p (Fig 7J). However, this band was present in 13 of the 17 individuals analyzed by C-banding, with 2 of them heterozygous for such band. An interstitial C-band in a same position was observed in one of six specimens of *B*. *caingua* studied with this technique (LGE 15114). This band was present in only one homologue of pair 1p, but was subtle (Fig 7K).

In the eight species analyzed with DAPI/CMA_3_, bright CMA_3_+ signals (DAPI-) were exclusively restricted to NOR sites. Additional bands of similar composition (DAPI-/CMA_3_+) were present in an interstitial position on pair 1p and in all centromeres in *Boana cipoensis*, and pericentromeric on pair 3p in *B*. *pulchella*. In the latter, AT-rich heterochromatin (DAPI+) was present on pericentric 6p (Fig 4, S2 Fig).

**Fig 8:**
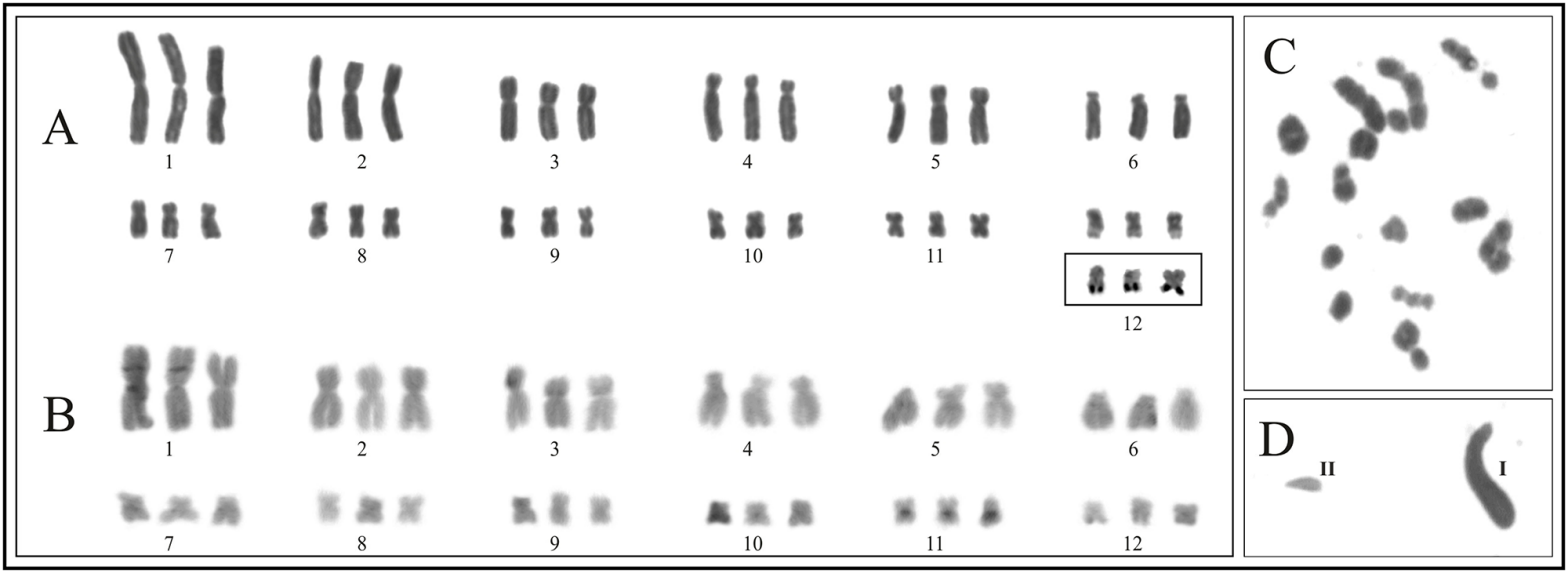
Triploid specimen of *Boana pulchella* (LGE 11504). **A**. Giemsa stained karyotype. **B.** C banded pattern. **C.** Meiotic cell in metaphase I stage. **D.** Normal spermatid (**I**) and abnormal microspermatid (**II**).

Remarkably, one specimen of *Boana pulchella* (LGE 11504) showed a triploid complement (2n = 3x = 36), with similar chromosome morphology, three Ag-NORs, and C-banding pattern with respect to the other specimens analyzed but with heterozygous interstitial band present in only two of three chromosomes 1 (Fig 8). The meiotic analysis shows the formation of trivalents (IIIs), bivalents (IIs), and univalents (Is) during the first meiotic stage and the presence of microspermatids (Fig 8C and 8D).

## Discussion

Two basic chromosome numbers are often observed in Hylidae, x = 12 and x = 13. Most species of Phyllomedusinae and Pelodryadinae karyotyped to date share an x = 13 ([23,26,50–56], and references therein), excepting *Nyctimystes infrafrenatus* (x = 12) [51].

In Hylinae, a basic number of x = 12 is widespread, being observed in ca. 80% species for which chromosome data are available (see Material and methods and S1 Table for references). This number is rare among other athesphatanurans, observed only in species of Dendrobatoidea: Aromobatidae and Dendrobatidae ([57], and references therein), and Leptodactylidae ([58–60], and references therein).

In Hylinae, deviations from the modal number x = 12 has been inferred as apomorphies of several lineages. In Lophyohylini, *Phyllodytes edelmoi* and *P*. *luteolus* share x = 11, *Osteopilus ocellatus* x = 17, and *O*. *wilderi* x = 14 [38,61]. Similarly, in Hylini the three species of *Acris* have x = 11 [35,62,63]. In Dendropsophini, the diploid number of x = 12 is also common, despite the derived condition of x = 15 observed in all the 33 studied species of *Dendropsophus* ([25,30,63–65], and references therein). Other variations within Dendropsophini correspond to *Pseudis cardosoi* (x = 14), *Scarthyla goinorum*, *Scinax constrictus* (x = 11), and *Sphaenorynchus carneus* (x = 13) [25,66,67].

In Cophomantini, distinct basic chromosome numbers are restricted to *Aplastodiscus*, *Hyloscirtus alytolylax* and some species of the *Boana albopunctata* group, discussed below [46,68,69]. Almost all *Aplastodiscus* species have a chromosome basic number lower than x = 12 (9, 10, and 11), that may be explained by the occurrence of two independent reduction events. Reduction from x = 12 to x = 11 occurs in species of the *A*. *albofrenatus* group, and from x = 12 to x = 10 and x = 9 in the *A*. *albosignatus* group; whereas both species of the *A*. *perviridis* group (*A*. *perviridis* and *A*. *cochranae*) share the plesiomorphic condition of x = 12 [8,46]. All species of *Bokermannohyla* show similar karyotypes regarding chromosome number (x = 12) and morphology [70].

Two karyotyped species of *Hyloscirtus* have x = 12 chromosomes but with some differences in their karyotypes (*H*. *larinopygion* with a FN = 46 and *H*. *palmeri* with FN = 48). In addition, Duellman et al. [71] mentioned that *H*. *armatus* (as *Hyla armata*) also has 24 chromosomes (figures not shown in that work), which supports the occurence of a plesiomorphic karyotype with 2x = 24 chromosomes in the three species groups of *Hyloscirtus*. The karyotype of x = 10 observed in *H*. *alytolylax* (FN = 38) represents another event of chromosome reduction in Cophomantini, and cytogenetic analysis of related species will establish the taxonomic distribution of this character in the genus.

Based on current phylogenetic knowledge [2,4–7], the karyotypes of *Hyloscirtus larinopygion* and *H*. *palmeri* provide new evidence to support the ancestral condition of x = 12 as a synapomorphy of Hylinae, as previously proposed [2,25,37], althought the karyotype of *Myersiohyla* is still unknown.

### Reductions in chromosome number in the *Boana albopunctata* group and the presence of B chromosomes

The composition of the *Boana albopunctata* group increased significantly in the last decade, from 9 to 14 species [13,72]. For this group, two diploid numbers are observed: *Boana almendarizae*, *B*. *calcarata*, *B*. *fasciata*, *B*. *heilprini*, and *B*. *raniceps* have karyotypes with 2n = 2x = 24, and *B*. *albopunctata*, *B*. *leucocheila*, and *B*. cf. *alfaroi* 2n = 2x = 22 ([31,48,67,73–75], this study). Bogart and Bogart [73] reported 2n = 2x = 24 chromosomes for a male of *B*. *fasciata* (as *Hyla fasciata*) collected in Bosque Nacional de Iparía (Huanuco, Perú). However, the putative occurrence of two species similar to *B*. *fasciata* (*B*. *calcarata* and *B*. *maculateralis*, [13]) has been reported recently. Hence, in the absence of a voucher specimen, the cytogenetic information provided for *B*. *fasciata* by Bogart and Bogart [73] should be considered with caution.

We observed discrepancies between our results and the literature for the karyotypes of *Boana multifasciata* and *B*. *lanciformis*. A karyotype of 2n = 2x = 24 was described for *B*. *multifasciata* in specimens from the Brazilian localities of Serranópolis, Goiás [67], and Iranduba, Amazonas [48]. On the contrary, we observed a karyotype of 2n = 2x = 22 for this species, in specimens collected near the type locality (Belém, State of Pará, Brazil). This species has been recovered in most phylogenetic hypotheses as a related taxon to *B*. *albopunctata* (i.e. [2,4,5,7,21]), which also has a standard karyotype of 2n = 2x = 22 and similar C-banding pattern and NORs location [63,74,75]. Based on the previous results, it is rather likely that *B*. *multifasciata* also has 2n = 2x = 22 chromosomes.

Two other taxa sharing this karyotype are *Boana leucocheila* and *B*. cf. *alfaroi* (from Carajas, state of Pará, Brazil). A number of 2n = 2x = 22 was also described for *B*. *lanciformis* from Manaus, State of Amazonas, Brazil [48], in contrast to our report of 2n = 2x = 24 for one specimen of *B*. cf. *lanciformis* from São Gabriel da Cachoeira, also State of Amazonas. Although the specimens studied by Mattos et al. [48] are morphologically similar to the one studied by us, it is likely that these chromosomal differences correspond to cryptic diversity, a taxonomic issue that merits future investigation.

It would be reasonable to assume that the karyotype of x = 11 with NORs on pair 8 observed in *Boana* cf. *alfaroi*, would be homologous to those observed in *B*. *albopunctata*, *B*. *leucocheila*, and *B*. *multifasciata*. Morphological traits of specimens studied here as *B*. cf. *alfaroi* (dark spotted pattern on the flanks and thighs, small tubercle on the heel, and small tympanum diameter), suggest that this taxon belongs to the *B*. *calcarata*-*B*. *fasciata* complexes (*sensu* Caminer and Ron [13]). In this sense, there are two issues that hinder the identification of the node or nodes where the reduction from x = 12 to x = 11 occurred. First, the lack of cytogenetic information for *B*. *dentei* and most species of the *B*. *calcarata- B*. *fasciata* complexes; and second, the absence of an inclusive phylogenetic analysis of the *B*. *albopunctata* group. The reduction in the chromosome number from 24 to 22, that occurs the *B*. *albopunctata* group, may have involved the smallest pairs of chromosomes, switching NORs from pair 11 in species with 2n = 24, to 8 in those with 2n = 22 [63,74].

*Boana albopunctata* shows two morphologically distinct B chromosomes: a medium-sized supernumerary [74], and a small-sized one [75], both with pericentromeric DAPI+ heterochromatin located on the short arm. We report the occurrence of another case of B chromosomes in two of three specimens of *B*. *leucocheila*, whose morphology and size resembles that found in argentine populations of *B*. *albopunctata* [75]. Although the phylogenetic position of *B*. *leucocheila* has not been assessed, this species is very similar to *B*. *albopunctata*, and these supernumerary elements could possibly have a single origin as a by-product of the chromosome reduction that occurred in these species.

Gruber et al. [76] performed chromosome painting on preparations of *Boana albopunctata* and *B*. *raniceps* using a chromosome probe obtained from the microdissection of a B chromosome of *B*. *albopunctata*. These authors observed exclusive hybridization on the B chromosomes of *B*. *albopunctata* and intercalary hybridization signals on all chromosomes of *B*. *raniceps*. Moreover, they also performed genomic hybridization (GISH) with genomic DNA of *B*. *raniceps* in B+ mitotic metaphases of *B*. *albopunctata*, observing signals on the B chromosome and, eventually, on pairs 3 and 8. Based on their results, Gruber et al. [76] proposed an interspecific origin of the B chromosome of *B*. *albopunctata*, but without explaining a possible mechanism. The transposition of sequences, originated mainly from regular chromosomes, could be closely related to the origin and evolution of B chomosomes [77–79], and could explain the hybridization amalgamation pattern obtained for *B*. *raniceps* when using the B-probes. Nonetheless, the hypothesis proposed by Gruber et al. [76] for an interspecific origin of the B chromosome in *B*. *albopunctata* is premature and needs further empirical support. Additional cytogenetic studies are still necessary to unveil the origin of the B chromosomes in both *B*. *albopunctata* and *B*. *leucocheila*, and their possible relation to the chromosome reduction observed in related species.

### Spontaneous polyploidy in *Boana pulchella*

Polyploidy is extremely rare among Hylidae as it is only known in *Phyllomedusa tetraploidea* (2n = 4x = 52) and *Hyla versicolor* (2n = 4x = 48; see [80] for a review). In this family, findings of odd allopolyploidy events (2n = 3x) in natural populations, may be due to hybridization between the tetraploid species *P*. *tetraploidea* and *H*. *versicolor*, with the closely related diploid species *P*. *distincta* [81] and *H*. *chrysoscelis* [82] respectively.

In Cophomantini, there are no records of polyploidy until the present report of a triploid specimen of *Boana pulchella* from the locality of Gobernador Virasoro (Corrientes, Argentina). This specimen was phenotypically indistinguishable from 37 diploid counterparts that were studied, including five other specimens from the same locality. This report is the first record of spontaneous autopolyploidy in a natural population of a diploid hylid species. This phenomenon is infrequent among anurans and has been observed only in eight species, *Amietophrynus poweri* (Bufonidae, [83]), *Engystomops coloradum* (Leptodactylidae, [84]), *Eupsophus vertebralis* (Alsodidae, [85]), *Holoaden luederwaldti* (Craugastoridae, [86]), *Leiopelma hochstetteri* (Leiopelmatidae, [87]), *Lithobates palustris* and *L*. *pipiens* (Ranidae, [88,89]), *Odontophrynus americanus* (Odontophrynidae, [90]), and *Xenopus tropicalis* (Pipidae, [80]). The ocurrence of univalents, bivalents, and trivalents in meiosis, as well as the presence of microspermatids, suggest that this specimen would have a reduced fertility due to the formation of unbalanced gametes.

### Informative variation in NORs location

The NOR location revealed by silver impregnation or FISH has proved to be an important marker for the study of the chromosome evolution in anurans (e.g. [24,28,69,91–93]). In Cophomantini, NORs located on small chromosomes are frequently observed in *Aplastodiscus*, *Boana*, and *Bokermannohyla*. Likewise, this is also frequent in members of the other tribes of Hylinae, suggesting that this condition would be widely distributed in the subfamily, and that small chromosomes carrying NORs might be homeologous [24,70].

In *Hyloscirtus*, the NOR positions show an interesting variation: *H*. *alytolylax* and *H*. *palmeri* share NORs in similar sized chromosomes (pair 4), although in very different locations (4q and 4p respectively), while *H*. *larinopygion* present NORs in pair 1. These conditions are observed homoplastically in a few other members of the tribe (see below). The interspecific variability observed in *Hyloscirtus* for the position of NORs plus the variation in 2n, FN, and other banding patterns, are features that likely will prove informative when more species of this genus are studied cytogenetically.

In most species of *Bokermannohyla* the NORs are located on both homologues of pair 11, with the exception of *B*. *alvarengai* and *B*. *ibitiguara* where they are on pairs 4 and 1, respectively [70]. In *Aplastodiscus* this marker was studied in all eight karyotyped species [46,68,69]. Both studied species of the *A*. *perviridis* group (*A*. *cochranae* and *A*. *perviridis*), and those of the *A*. *albosignatus* group (*A*. *albosignatus*, *A*. *callipygius*, and *A*. *leucopygius*) share the plesiomorphic condition of NORs on a small-sized chromosome (pairs 11 and 9 respectively, but apparently homeologous). Within the *A*. *albofrenatus* group, NORs occur on pair 11 in *A*. *arildae* and on medium-sized chromosomes (6 or 7, but apparently homeologous) in *A*. *ehrhardti*, *A*. *eugenioi*, and *A*. *albofrenatus*. The optimization of this character on the most inclusive phylogenies for *Aplastodiscus* [7,8] has led to an ambiguity at the base of the *A*. *albofrenatus* group, due to the position of *A*. *arildae* (see S1 Fig).

In *Boana*, 25 of 39 species present interstitial or terminal Ag-NORs on the long arm of the smallest chromosomes (10 to 12). Exceptions are some species of the *B*. *albopunctata*, *B*. *pulchella*, and *B*. *semilineata* groups ([48,49,74,94–97], this study). In most species NORs are on a single pair of homomorphic chromosomes, a condition suggested as the ancestral state for Anura [98]. The only report of multiple NORs corresponds to *B*. *atlantica*, on both homologues of pairs 10 and 12 [49]. Although *B*. *prasina* shows more than one pair of NORs (i.e. on 9 and 12), and it is a polymorphic condition [94]. Other variations associated with the NORs were observed in the size of Ag-NORs between homologues in *B*. *albomarginata*, *B*. *faber*, *B*. *semilineata*, and *B*. *punctata* ([68,97], this study). Heteromorphic size of Ag-NORs and secondary constrictions are a very frequent feature among anurans [99].

In the *Boana semilineata* group, NORs are interstitial on pair 7 in *B*. cf. *semilineata* (this study), *B*. *pombali* [49], and *B*. *semilineata* [49,97], centromeric on pair 1 in *B*. *geographica* [48], and terminal on pair 11 in *B*. *wavrini* ([48], this study). The NOR location in *B*. *boans*, has been reported on pair 7 ([56], this study) or pair 11 [48]. However, given the morphological similarity between *B*. *boans* and *B*. *wavrini* [100], these differences would likely correspond to species misidentification. It is likely that NORs on pair 7 represent a synapomorphy of a less inclusive clade within the group, whose limits will be inferred when more information on karyotypes and phylogenetic relationships of the group become available by further data (only *B*. *boans*, *B*. *geographica*, and *B*. *semilineata* have been included in phylogenetic studies).

In most species of the *Boana pulchella* group, NORs are located on pair 11. However, NORs occur on pair 1 in *B*. *curupi* (as *Hyla* aff. *semiguttata* in [96], this study), *B*. *cipoensis* (this study), *B*. *joaquini* (as *Hyla semiguttata* from the localities of Cambará do Sul and São Francisco de Paula, Brazil, [96]; Paulo Pinheiro pers.com.), *B*. *semiguttata* [96], and *B*. *stellae* (this study). Some of these species (*B*. *curupi*, *B*. *joaquini*, *B*. *semiguttata*, and *B*. *stellae*) are part of a monophyletic group restricted to the Atlantic Forest, the *B*. *semiguttata* clade ([10,11,101], Faivovich, pers. obs.). *Boana cipoensis*, on the other hand, belongs to the *B*. *polytaenia* clade ([4], Faivovich pers. obs.), the sister taxon of the *B*. *semiguttata* clade. Although NOR location should be investigated in the remaining members of these clades, its presence on pair 1 is a putative synapomorphy supporting the monophyly of the *B*. *polytaenia* and *B*. *semiguttata* clades, so far supported only by molecular data ([11], and references therein). Other three species of the *B*. *pulchella* group (*B*. *caingua*, *B*. *prasina*, and *B*. *pulchella*) share NORs on pair 12, suggesting that this condition represents a less inclusive synapomorphy within the *B*. *pulchella* group (see Fig 4 and S1 Fig).

### Heterochromatin patterns

The high variability of heterochromatin, consequence of both its evolutionary dynamics and the general lack of knowledge about its composition make it difficult to establish the homology of heterochromatic bands among related taxa. In this sense, heterochromatin should be used with caution in phylogenetic studies [102]. Nevertheless, C-banding patterns have been shown to be phylogenetically informative for some groups (e.g. [24,25,59,93,103,104]).

Heterochromatin in most of species of Cophomantini is mainly restricted to centromeres, secondary constrictions (NOR sites), or proximally to these structures, as in most anurans [105]. C-bands are known for 53 species of Cophomantini: 8 species of *Aplastodiscus*, 33 species of *Boana*, 9 species of *Bokermannohyla*, and 3 species of *Hyloscirtus* ([46,49,69,70,74,94–96], this study). The better-sampled taxa show interesting features to be discussed.

In the *Boana pulchella* group a conspicuous interstitial heterochromatic C-band is present in one of the smallest chromosomes in the karyotype of most species (pair 11), with the exception of *B*. *prasina* [94], *B*. *cipoensis*, and *B*. *marianitae* (this study). Sequential C-bands and Ag-NORs confirmed that C-bands co-occur with NORs in *B*. *albonigra*, *B*. *bischoffi*, *B*. *cordobae*, *B*. *marginata*, *B*. *guentheri*, and *B*. *riojana*, but not in *B*. *caingua* and *B*. *pulchella* that have NORs in pair 12 (see above). Chromosomes bearing this band were previously considered as pair 10 in *B*. *bischoffi*, *B*. *guentheri* [95], *B*. *curupi*, *B*. *joaquini*, *B*. *marginata*, and *B*. *semiguttata* [96], indicating differences in the ordering criteria of the chromosomes. This result suggests the possible homology of the elements carrying this C-band among all species, and probably represents a synapomorphy for the *B*. *pulchella* group, or a less inclusive clade, pending the characterization of chromosomes of the earlier diverging species of this group.

We observed a conspicuous C-band on 6p in *Boana curupi*, absent in *B*. *stellae*. Interestingly, Ananias et al. [96] did not report any difference in the heterochromatin pattern of pair 6 between *B*. *curupi*, *B*. *joaquini*, and *B*. *semiguttata*; however, in the ideogram of their paper ([96]: fig. 4), represented centromeres of *B*. *curupi* and *B*. *semiguttata* have higher amount of heterochromatin than in *B*. *joaquini*. This might be a cytogenetic character of taxonomic importance and, similarly to NORs on pair 1 (see above), it is important to determine its taxonomic distribution in the *B*. *semiguttata* clade.

Finally, we detected interstitial heterochromatin on pair 1 of *Boana pulchella* and *B*. *caingua*. In the first species, this C-band was conspicuous in 13 of 17 specimens (11 homozygous and two heterozygous, one of them triploid), and in the second, it was a subtle heteromorphic band only detected in one of six specimens studied with this technique. The polymorphic bands observed in both species had no association with sex and would explain why Baraquet et al. [106] did not detect it on *B*. *pulchella*. Instead, these authors emphasized a heterochromatic band with similar location in *B*. *cordobae*, not observed here for that species. Interestingly, a heterochromatic band in an analogous position is also present in *B*. *prasina* [94]. In this context, this heterochromatic band may represent a homologous polymorphic band present in *B*. *caingua*, *B*. *cordobae*, *B*. *prasina*, and *B*. *pulchella*, which deserves further attention.

Additional variation was observed in the smaller chromosomes of *Boana punctata* karyotype (i.e. 11 and 12), regarding morphology and heterochromatin. Unfortunately, for this species, we studied only two females, and in previous descriptions only reported the chromosomal number [63,73] and the pattern of C-bands as an ideogram representation [35]. Therefore, it would be necessary to study a larger number of specimens of both sexes, in order to better understand the occurrence of these heteromorphisms.

The fluorochromes DAPI and CMA_3_ have proved to be uninformative for most species of *Aplastodicus*, *Boana*, and *Hyloscirtus*. In *Bokemannohyla*, however, all 10 species studied with this technique showed a similar pattern of CG-rich (CMA_3_+) centromeric heterochromatin [70], which represents an interesting characteristic for study in the genus. For most species of *Boana* and *Hyloscirtus* analyzed here it was not possible to detect a common pattern that would represent a possible phylogenetic signal, as fluorescent bands varied in quantity and composition, with the exception of the general feature of DAPI-/CMA_3_+ signals detected in NOR sites. Other signals were rarely observed and represent peculiarities, in most cases associated to centromeric, pericentromeric, and telomeric heterochromatic regions. In most studied species centromeres do not show differences for either DAPI or CMA_3_ fluorochromes; in others centromeres are DAPI+, in a few exceptions DAPI-/CMA_3_+, and only in *B*. cf. *semilineata* DAPI+/CMA_3_-. Exceptional cases of variation in fluorescent patterns were seen in the three species of the *B*. *semilineata* group, *B*. *boans*, *B*. cf. *semilineata*, and *B*. *wavrini*, each one with a particular pattern ([56], this study). Interestingly, some chromosomes of *B*. *boans*, *B*. *geographica*, and *B*. *wavrini* have interstitial telomeric sequences (ITSs) [48,56]. ITSs are internal or intrachromosomal telomeric sequences that may arise through different rearrangements, that have been reported for 43 anurans (see [56] for a review).

In Cophomantini, the location of telomeric DNA has been mapped by FISH in some species of *Aplastodiscus* and *Boana* [46,48,56,69,75]. In *Aplastodiscus* ITSs were reported in *A*. *albofrenatus*, *A*. *arildae*, *A*. *eugenioi* (x = 11), and *A*. *leucopygius* (x = 9), absent in *A*. *albosignatus* (x = 10), *A*. *ehrhardti* (x = 11), and *A*. *perviridis* (x = 12), although their presence would not be related to the chromosome reduction that occurred in this group [46,69]. Moreover, in *Boana*, ITSs were detected in three species of the *B*. *semilineata* group (*B*. *boans*, *B*. *geographica*, and *B*. *wavrini*), and *B*. *faber* [48,56], but were absent in *B*. *albopunctata* [75], *B*. *cinerascens*, *B*. *lanciformis*, *B*. *multifasciata*, and *B*. *raniceps* ([48], data not shown in that work).

In the species of the *Boana semilineata* group two kinds of ITSs were detected; those restricted to heterochromatic regions and a rare type of large ITSs restricted to euchromatic regions (eu-ITSs), which are rare among vertebrates [56]. Heterochromatic ITSs were observed in a centromeric position of pairs 1 and 5 of *B*. *geographica*, and interstitially on the long arm of pair 5 of *B*. *boans* and *B*. *wavrini*, whereas eu-ITSs were reported on the short arm of pair 2 of *B*. *boans* and *B*. *wavrini* [48,56]. Schmid and Steinlein [56] described one additional conspicuous eu-ITS for pair 9 of *B*. *boans*, not previously detected by Mattos et al. [48]. The authors highlighted this feature but did not mention a different morphology of pair 5 with respect to the karyotype analyzed by Mattos et al. [48], probably because of differential chromosome ordering criteria for pairs 3–5. Our results agree with Schmid and Steinlein [56], since the specimens of *B*. *boans* we analyzed have an evidently higher centromeric index, as compared to that described by Mattos et al. [48]. Curiously, we observed an important difference in the morphology of pair 5 between *B*. *boans* (CI: 0. 36) and *B*. *wavrini* (near subtelocentric, CI: 0. 25).

It is likely that the karyotypes of *Boana boans* and *B*. *wavrini* would be distinguishable by the position of NORs, number of ITSs, and the morphology of pair 5. The morphological similarities between these species may have caused misidentification by Mattos et al. [48]. Moreover, among all species studied for the *B*. *semilineata* group ([48,49], this study), pair 5 with a lower centromeric index is an apparently unique feature of *B*. *wavrini*.

It is worth mentioning the heteromorphism observed for pair 8 in one specimen of *Boana boans*, with one submetacentric chromosome (8A) and another metacentric (8B). This heteromorphism was detected on a single male (PS 785). The different arm ratio between both chromosomes 8A and 8B is a consequence of a longer short arm of pair 8B, and the absence of constitutive heterochromatin. Interestingly, as it was mentioned above, eu-ITSs occur on the short arms of pairs 2 and 9 of this species [56]. Taking this into account, and the possibility that ITSs could disperse through the genome due to the proximity of non-homologous chromosomes with similar shape [107], it is tempting to propose the presence of eu-ITSs on the short arm of 8B as a possible cause of this heteromorphism. However, it is still necessary to confirm this hypothesis by performing telomeric FISH.

### Sex chromosome heteromorphism in *Hyloscirtus*

Among vertebrates, anurans are a group that has an interesting plasticity for genetic determination of sex, which can be through differentiated heteromorphic sex chromosomes (XY and ZW), or by sex chromosomes that are microscopically indistinguishible (homomorphic). Moreover, extreme examples of variability for sex chromosome determination systems are observed in the *Glandirana rugosa* complex [108,109] and *Leiopelma hochstetteri* [110]. In the *G*. *rugosa* complex, systems can vary between populations, as heterogamety is observed in both males (XY) or females (ZW), although it may also be absent, without differentiated chromosomes in either sex. In *L*. *hochstetteri* sex is determined by a variable W univalent present in females, showing a chromosomal sex determination unique among vertebrates (W0 females/00 males). Regardless of the different ways of determining sex in anurans, the presence of identifiable sex chromosomes is an infrequent phenomenom found only in approximately 40 species, where most of the time sex-determining chromosome identification is only possible with chromosome-banding techniques ([32], and references therein; [111]).

In Hylinae, the cytogenetic demonstration of sex chromosomes is sparse, being reported in *Hyla immaculata* and *Pseudis tocantins* as ZZ/ZW sytems ([34,36], and references therein), whereas *H*. *femoralis* shows a XX/XY system [35,112]. Other cytogenetic observations on *H*. *japonica* and *H*. *squirella* provide evidence of the presence of XY and ZW sex chromosomes, respectively. First, a different meiotic behaviour of pair 1 was observed during meiosis I in *H*. *japonica*, and second, the presence of a heterochromatic C-band on one of the homologous chromosomes of pair 1 in females of *H*. *squirella* was observed (see [35] for a review). Further molecular studies on the *Hyla arborea* group have also confirmed male heterogamety (i.e. XY) in *H*. *arborea*, *H*. *orientalis*, *H*. *intermedia*, *H*. *meridionalis*, *H*. *molleri* and *H*. *sarda* [113–116].

We observed a heteromorphic banding pattern with the fluorochrome DAPI for pair 2 in *Hyloscirtus alytolylax*, accompanied with slight differences in size. In the three studied males, the centromere of one of the homologous of pair 2 has bright DAPI+ heterochromatin, whereas in females none of the chromosomes of this pair showed these, indicating that the males were heterogametic (XX/XY). The overall absence of heterochromatin on 2Y, in addition to the small size differences with its counterpart (2X), are not sufficient evidence to suppose a recent origin of this system, as sex chromosomes diferentiation could be delayed by a rapid change of sex determining genes (turnovers) and recombination between heteromorphic chromosomes through occasional sex reversion [114,116,117].

The presence of sex chromosomes in the particular karyotype of *Hyloscirtus alytolylax* (i.e. 2n = 20, FN = 38), unique within Cophomantini, and the lack of information in other species, precludes us from supposing the possible chromosome(s) or rearrangement(s) that have participated in its origin. In addition, because we have only analyzed one female *H*. *palmeri*, we do not know if this characteristic is also present in this species or others of the *H*. *bogotensis* group.

## Conclusions

In Cophomantini, a basic number of x = 12 is rather frequent, but chromosome number reduction is present at least in four independent lineages. These reductions likely have played an important role in chromosome evolution of some groups, raising the future challenge of detecting the possible chromosomes involved and the rearrangements that occurred. Most species share NORs on small-sized chromosomes, suggesting a putative homology between chromosomes carrying this marker. Variation in this character provides valuable phylogenetic evidence in some groups (e.g., the *Boana albopunctata*, *B*. *pulchella*, and *B*. *semilineata* groups). Heterochromatin, on the other hand, represents a potential source of information that should be studied further. For instance, the presence and location of ITSs and the putative homology between some heterochromatic bands needs investigation. Sex and B chromosomes also contribute to variability in cophomantines and, despite their infrequent occurrence, represent another interesting feature that deserves to be explored as they can provide important information about past evolutionary processes. Finally, the presence of x = 12 chromosomes in *Hyloscirtus* supports the hypothesis that this character state represents a synapomorphy for Hylinae. Nonetheless, it is crucial to determine the chromosome number of *Myersiohyla*, since the presence of the number of x = 13 in the genus would turn ambiguous the optimization of this character in the ingroup node of Hylinae.

## Supporting information

S1 File Information of the specimens analyzed of each species. (PDF)

S1 Table Cytogenetic information in Cophomantini. Differential techniques performed, chromosome number (2n) and NORs position observed in each species.
(PDF)

S2 Table Chromosome measurements of 25 species of *Boana* and 3 of *Hyloscirtus*. (a) *B*. *albopunctata* group; (b) *B*. *faber group*; (c) *B*. *pellucens* group; (d) *B*. *pulchella* group; (e) *B*. *punctata* group; (f) *B*. *semilineata* group. ^(B)^ B chromosome in *B*. *leucocheila*. Chromosome percentage relative to the haploid set (Chromosome Mophology) Centromeric Index ± Standard Deviation. m: metacentric; sm: submetacentric; st: subtelocentric; t: telocentric.
(PDF)

S1 Fig Optimization of the basic number (X) and the position of NORs (NORs) in Cophomantini on the phylogenetic hypothesis of Duellman et al. (2016). (PDF)

S2 Fig DAPI and CMA_3_ staining in 20 species of *Boana*. The square shows heteromorphic NOR-bearing chromosome pair in *B*. *punctata* sequentially stained by the silver impregnation technique.
(TIF)
