# Supplementary material for: Chromosome evolution in Cophomantini (Amphibia, Anura, Hylinae)
Source: PLoS One. 2018 Feb 14;13(2):e0192861. doi: 10.1371/journal.pone.0192861 (PMC5812657; doi:10.1371/journal.pone.0192861)
Supplement: S1 File — (PDF) [file pone.0192861.s001.pdf]

S1 File. Information of the specimens analyzed of each species.

Collected specimens are housed in the herpetological collections of Laboratorio de Genética Evolutiva, Instituto de Biología Subtropical, Posadas, Misiones, Argentina (LGE), Museo de La Plata, La Plata, Buenos Aires, Argentina (MLP), Museo Argentino de Ciencias Naturales "Bernardino Rivadavia", Buenos Aires, Argentina (MACN), Centro de Estudos Avançados da Biodiversidade, Universidade Federal do Pará, Belém, Pará, Brazil (PS), Museu Paraense Emílio Goeldi, Pará, Brazil (MPEG), Museo de Zoología de la Pontificia Universidad Católica del Ecuador (QCAZ), Coleção Herpetológica do Departamento de Zoologia, Universidade Federal do Paraná, Curitiba, Paraná, Brazil (DZUP), Departamento de Zoologia, Instituto de Biociências, UNESP, Rio Claro, São Paulo, Brazil (CFBH), Museu de Zoologia da Universidade Estadual de Campinas "Adão José Cardoso", Campinas, São Paulo, Brazil (ZUEC), Museo Nacional de Historia Natural, Montevideo, Uruguay (MNHN), Dr. Diego Baldo field number (DB), Dr. Martín Oscar Pereyra field number (MP). Male (M), female (F), juvenile (J), undetermined (U).

Hyloscirtus larinopygion — ECUADOR: Carchi: Morán, QCAZ 55574 (M), 55575 (M).

Hyloscirtus palmeri — ECUADOR: Esmeraldas: Durango, Finca Sr. Antonio, QCAZ 51852 (F).

Hyloscirtus alytolylax — ECUADOR: El Oro: Reserva Biológica Buenaventura, QCAZ 68444 (M), 68445 (M), 68446 (F), 68447 (F), 68448 (M).

Boana almendarizae — ECUADOR: Tungurahua: Río Verde, QCAZ 51900 (M); Colonia Azuay, QCAZ 52020 (M).

Boana calcarata — ECUADOR: Pastaza: Centro Etnoturístico Indichuris; QCAZ 62868 (M).

Boana cf. *lanciformis* — BRAZIL: Amazonas: São Gabriel da Cachoeira, CFBH 39717 (M).

Boana cf. *alfaroi* — BRAZIL: Paraupebas: Carajas, Dique, PS 597 (M), MPEG 40198 (U).

Boana heilprini — Pet trade: H10 (F), H12 (M)

Boana leucocheila — BRAZIL: Pará: Juruti, Acampamento Barroso, MPEG 22210 (M); Mutúm, MPEG 22211 (M); Alcoa, MPEG 22394 (M).

Boana multifasciata — BRAZIL: Pará: Igarapé-Miri, Vila Maiauata PS 18 (M), 34 (M); Curuçá, Ilha Pedras Grandes, Igarapé do Pinico, PS 692 (M), 693 (U).

Boana raniceps — ARGENTINA: Misiones: Capital, Villa Blosset, LGE 11605 (M), 11678 (M), 11685 (M), 11692 (M), 11707 (M), 11711 (F); Posadas, Camping municipal El Brete, LGE 11714 (M); Garupá, LGE 11724 (M). Chaco: San Fernando, Ruta Nacional 11, 1 km south from Basail, LGE 11620 (M), 11622 (M); General Güemes, 15.5 km northwest from Misión Nueva Pompeya, LGE 11631 (J). Salta: Orán, Ruta Provincial 5, near Pichanal, LGE 11728 (M). Corrientes: San Miguel, Estero Carambola, Estancia Santa Julia, LGE 2021 (M); BRAZIL: Piauí: PNSC-PI, PS 81(U); Nazaré, PS 91 (U); Pará: Soure, PS 538 (M), 539 (M), 545 (M).

Boana faber — ARGENTINA: Misiones: Cainguas, Aristóbulo del Valle, Balneario Arroyo Cuñá Pirú, LGE 15112 (M); San Pedro, Tobuna, Ruta Provincial 224, 28 km from Salto Alegría, LGE 18128 (M), 18130 (M).

Boana pellucens — ECUADOR: Esmeraldas: Durango, Finca Sr. Antonio, QCAZ 51870 (M); Muisne - Cabo San Francisco, Laguna del Diablo, QCAZ 55576 (M).

Boana albonigra — ARGENTINA: Jujuy: Santa Catalina, Santa Catalina, Río Santa Catalina, LGE 3695 (F), LGE 17700 (M); Santa Catalina, El Queñoal, LGE 17732 (M).

Boana caingua — ARGENTINA: Misiones: Capital, Villa Lanús, Campus Universidad Nacional de Misiones, LGE 15113 (M), 17828 (M), 17832 (M), 17888 (M); 17907 (M), 17909 (M), 17910 (M), 17911 (M), 17912 (M), 17913 (M), 17979 (M); Capital, Garupá, Arroyo Garupá and Ruta Nacional 12, LGE 17833 (F); Candelaria, Candelaria, LGE 15114 (M).

Boana cipoensis — BRAZIL: Minas Gerais: Serra do Cipó, PS 905 (M); Diamantina, 500-1 (M), 500-2 (M), 500-3 (M).

Boana cordobae — ARGENTNINA: Córdoba: Colón, Rio Ceballos, Quebrada del Cóndor, LGE 11978 (M), San Luis, Coronel Pringles, near Inti Huasi, LGE 11956 (M), 11957 (M), 11958 (M), 11959 (F), 11960 (M), 11962 (M), 11963 (M), 11966 (M), 11967 (F),11968 (M).

Boana curupi — ARGENTINA: Misiones, Gral. Manuel Belgrano, Península Andresito, Arroyo Yacuy, LGE 18039 (M), 18041 (M), 18042 (M), 18044 (M), 18045 (M), 18046 (M).

Boana bischoffi — BRAZIL: São Paulo, Apiaí, Morro do Ouro, DZUP 356 (M).

Boana marianitae — ARGENTINA: Salta: Santa Victoria, Quebrada "La Cortadera", between Baritú and Río Sidras, Parque Nacional Baritú, MACN 50399 (M), MP 103 (M).

Boana pulchella — URUGUAY: Rocha, Laguna Negra, MNHN 11517 (M); Flores, 40 km south from Trinidad, MNHN 11518 (F); San José, Libertad, Facultad Veterinaria, MNHN 11520 (F); 11521 (M), 11525 (M). ARGENTINA: Buenos Aires: Tandil, Sierra de Animas, DB 2261 (M); Luján, Luján, Ruta Provincial 47, 11391 (M), LGE 11395 (M), 11398 (M), 11459 (M), 11475 (M), 11476 (M); Ensenada, La Plata, MLP 3944 (M). Misiones: Candelaria, Santa Ana, LGE 11403 (M), 11500 (M); Profundidad, LGE 11411 (M), 11423 (M), 11455 (M), 11462 (M), 11463 (M); Cerro Corá, LGE 11428 (M), 11429 (M),11472 (M), 11503 (M); Campo San Juan, LGE 11467 (M), 11493 (M); Apóstoles, Ruta Provincial 105, 3 km north from San José, LGE 11420 (M), 11440 (M), 11498 (M). Entre Ríos: Ruta Nacional 12 between Brazo Largo and Arroyo Don Luciano, Islas del Ibicuy, LGE 11430 (J). Corrientes: Santo Tomé, Gobernador Virasoro, LGE 6335 (M), 6336 (M), 6337 (M), 11413 (M), 11504 (M); Monte Caseros, Ruta Nacional 14, 23 km from intersection with Ruta Provincial 25, LGE 6403 (M); Curuzú Cuatiá, Perugorria, Estancia El Oscuro, LGE 19763 (F). San Luis: Juan Martín de Pueyrredón, El Volcán, La Hoya, LGE 9202 (M).

Boana riojana — ARGENTINA: La Rioja: Chilecito, Ruta Nacional 40, km 527, near El Siciliano, LGE 18016 (M), 18018 (M), 18021 (F), 18022 (M), 18036 (F); Famatina, Cuesta la aguadita, Ruta 11 between km 36-37, MLP 3381 (M), 3882 (M). Salta: Rosario de Lerma, Encón Grande, LGE 11944 (M). Catamarca: El Alto, Sierras de Ancasti, Rosario de Abajo, LGE 18028 (M); Guayamba, Río Guayamba, LGE 18032 (F). Tucumán: Tafí del Valle, Quebrada Zanja de los Cardones, Ruta Provincial 307, km 98, LGE 11952 (F), LGE 11953 (F), 11955 (F); Camping Los Sauzales, LGE 11951 (M).

Boana stellae — ARGENTINA: Misiones: Cainguas, Aristóbulo del Valle, Camping Arroyo Cuña Pirú, LGE 6500 (M), 14115 (M), 18090 (M), 18091 (M), 19583 (M), 19584

(M), 19585 (M), 19587 (M). Parque Provincial Salto Encantado, Salto Encantado, LGE 18098 (F).

Boana cinerascens — ECUADOR: Tungurahua: Rio Negro, QCAZ 51938 (M), 51941 (M), 52029 (M); BRAZIL: Pará: Santa Barbara do Pará, Gunma, PS 425 (M); Curuçá, Nazaré de Mocajuba, PS 823 (M), PS 824 (U); Boa Vista do Muriá, PS 835 (M). Amazonas, São Gabriel da Cachoeira CFBH 39716 (M).

Boana punctata — BRAZIL: Pará: Marabá, Marabá, PS 855 (F); Maranhão: Rio Itaueiras, Municipality of Porto Franco, CFBH 39626 (F).

Boana boans — BRAZIL: Pará: Melgaço, Caxiuana, PS 124 (U); Carajás, PS 785 (M); Marabá, Piçarreira MPEG 28137 (M).

Boana cf. *semilineata* — BRAZIL: Pará: Juruti, Mutúm, PS 510 (M); Marabá, Marabá, MPEG 28139 (U).

Boana wavrini — BRAZIL: Pará: Melgaço, Caxiuana, PS 123 (U), PS 127 (U), PS 181 (M).
