# Supplementary material for: Chromosome evolution in Cophomantini (Amphibia, Anura, Hylinae)
Source: PLoS One. 2018 Feb 14;13(2):e0192861. doi: 10.1371/journal.pone.0192861 (PMC5812657; doi:10.1371/journal.pone.0192861)
Supplement: S1 Table — Differential techniques performed, chromosome number (2n) and NORs position observed in each species. (PDF) [file pone.0192861.s002.pdf]

S1 Table. Cytogenetic information in Cophomantini. Differential techniques performed, chromosome number (2n) and NORs position observed in each species.

Genus	Species Group	Species	Reference	2n	Differential Techniques	NORs position
Aplastodiscus	*A. albofrenatus*	*A. albofrenatus*	Carvalho et al., 2009a	22	Ag-NORs, C-bands, FISH(rDNA, tel)	ter 7q(1)
		A. arildae	Bogart, 1973 (as *Hyla albofranata* from Boracéia, State of São Paulo, Brazil; according to Carvalho et al., 2009a)	22		
			Carvalho, 2009a	22	C-bands, FISH(rDNA, tel)	ter 10q
			Gruber et al. 2012	22	Ag-NORs, C-bands, FISH(rDNA, tel), BrdU, CMA3	ter 11q
		A. ehrhardti	Carvalho, 2009a	22	C-bands, FISH(rDNA, tel)	ter 6q(2)
		A. eugenioi	Carvalho, 2009a	22	C-bands, FISH(rDNA, tel)	ter 7q
			Gruber et al. 2012	22	Ag-NORs, C-bands, FISH(rDNA)	ter 6q
	A. albosignatus	*A. albosignatus*	Bogart, 1973	20		
			Carvalho et al 2009b	20	Ag-NORs, FISH(rDNA)	ter 9q
			Gruber et al 2012 (as *A. callipygius*)	20	Ag-NORs, C-bands, FISH(rDNA, tel), BrdU, CMA3(ns)	ter 9q
	A. albosignatus	*A. leucopygius*	Bogart, 1973 (as *Hyla albosignata* from Teresópolis State of Rio de Janeiro, Brazil; according to Carvalho et al., 2009b)	18		
			Carvalho et al 2009b	18	Ag-NORs, FISH(rDNA)	ter 9q
			Gruber et al. 2012	18	Ag-NORs, C-bands, FISH(rDNA, tel), BrdU, CMA3(ns)	ter 9q
	A. perviridis	*A. cochranae*	Carvalho et al. 2009b	24	Ag-NORs, C-bands, FISH(rDNA)	ter 12q
	A. perviridis	*A. perviridis*	Carvalho et al. 2009b	24	Ag-NORs, C-bands, FISH(rDNA)	ter 12q
			Gruber et al 2012	24	Ag-NORs, C-bands, FISH(rDNA, tel), BrdU, CMA3	ter 11q
Bokermannohyla	*B. circumdata*	*B. ahenea*	Catroli et al., 2011	24	Ag-NORs, C-bands, DAPI/CMA3(ns), BrdU	ter 11q
		B. circumdata	Catroli et al., 2011	24	Ag-NORs, C-bands, DAPI/CMA3, BrdU	ter 11q
		B. hylax	Catroli et al., 2011	24	Ag-NORs	ter 11q
		B. luctuosa	Baldissera Jr et al., 1993 (as *Hyla* sp. aff. *circumdata*)	24(B)	Ag-NORs	ter 11q
			Catroli et al., 2011	24	Ag-NORs, C-bands, DAPI/CMA3	ter 11q
		B. ravida	Catroli et al., 2011 (as *Bokermannohyla* sp. 1)	24	Ag-NORs, DAPI/CMA3(ns)	ter 11q
		B. sp. 2	Catroli et al., 2011	24	Ag-NORs, C-bands, DAPI/CMA3(ns)	ter 11q
		B. sp. 3	Catroli et al., 2011	24	Ag-NORs, C-bands, DAPI/CMA3(ns)	ter 11q
		B. sp. 4	Catroli et al., 2011	24	Ag-NORs, DAPI/CMA3(ns)	ter 11q
	B. pseudopseudis	*B. alvarengai*	Catroli et al. 2011	24	Ag-NORs, C-bands, BrdU	ter 4p
		B. ibitiguara	Catroli et al. 2011	24	Ag-NORs, C-bands, DAPI/CMA3(ns)	ter 1q
		B. saxicola	Catroli et al. 2011	24	Ag-NORs, C-bands, DAPI/CMA3	ter 11q
	B. martinsi	*B. martinsi*	Catroli et al. 2011	24	Ag-NORs, C-bands, DAPI/CMA3	ter 11q
Boana	*B. albopunctata*	*B. albopunctata*	Beçak, 1968	22		
			Bogart, 1973	22		
			de Oliveira et al., 2012	22		
			Gruber et al., 2007	22(B)	Ag-NORs, C-bands, DAPI/CMA3, BrdU	ter 8p
			Ferro et al. 2012	22(B)	Ag-NORs, C-bands, DAPI/CMA3, FISH(rDNA, tel)	ter 8p
			Gruber et al., 2014	22(B)	BrdU, FISH(B-probe), GISH	
		B. cf. *alfaroi*	present study	22	FISH(rDNA)	
		B. almendarizae	present study	24	Ag-NORs, C-bands, DAPI/CMA3	
		B. calcarata	present study	24	C-bands	
		B. fasciata	Bogart and Bogart, 1971	24		
		B. leucocheila	present study	22(B)	Ag-NORs, FISH(rDNA)	ter 8p
		B. lanciformis	Mattos et al., 2014	22	Ag-NORs, C-bands, FISH(tel)(ns)	ter 11q
		B. cf. *lanciformis*	present study	24	Ag-NORs	ter 11q
		B. multifasciata	de Oliveira et al., 2012	24		
			Mattos et al., 2014	24	Ag-NORs, C-bands, FISH(tel)(ns)	ter 11q
			present study	22	Ag-NORs, C-bands, DAPI/CMA3, FISH(rDNA)	ter 8p
		B. heilprini	present study	24	DAPI/CMA3	ter 11q(4)
		B. raniceps	Rabello, 1970	24		
			Rabello et al., 1971	24		
			de Oliveira et al., 2012	24		
			Gruber et al., 2007	24	Ag-NORs, C-bands, BrdU	ter 11q
			Mattos et al. 2014	24	Ag-NORs, C-bands, FISH(tel)(ns)	ter 11q
			Gruber et al., 2014	24	FISH(B-probe)	

			present study	24	Ag-NORs, C-bands, DAPI/CMA3	ter 11q
	B. faber	***B. albomarginata***	Beçak, 1968	**24**		
			Bogart, 1973 (as *Hyla albofrenata* from Tijuca Forest, State of Rio de Janeiro)	**24**		
			Nunes and Fagundes, 2008a	**24**	**Ag-NORs**	**int 2p**
			Carvalho et al 2009b	**24**	**Ag-NORs, C-bands, FISH(rDNA)**	**int 2p**
		B. crepitans	Duellman and Cole, 1965	**24**		
			Rabello, 1970	**24**		**int 6p(sc)**
			Bogart, 1973	**24**		
			Gruber et al., 2007	**24**	**Ag-NORs, C-bands, DAPI/CMA3(ns), BrdU**	**int 11q**
			Carvalho et al. 2014	**24**	**Ag-NORs, C-bands**	**int 7q**
		B. faber	Beçak, 1968	**24**		
			Nunes and Fagundes, 2008a	**24**	**Ag-NORs**	**ter 11q**
			Carvalho et al., 2009b	**24**	**Ag-NORs, FISH(rDNA)**	**ter 11q**
			Schmid and Steinlein 2016a	**24**	**Ag-NORs, C-bands, DAPI/DA/Myt, QM, H, FISH(tel)**	**ter 11q**
			present study	**24**	**Ag-NORs, C-bands, DAPI/CMA3, FISH(rDNA)**	**ter 11q**
		B. lundii	de Oliveira et al. 2012	**24**		
		B. pardalis	Bogart, 1973	**24**		
			Nunes and Fagundes, 2008a	**24**	**Ag-NORs, FISH(rDNA)**	**ter 11p**
		B. rosenbergi	León, 1970	**24(ns)**		
	B. pellucens	***B. pellucens***	present study	**24**	**Ag-NORs, C-bands, DAPI/CMA3, FISH(rDNA)**	
		B. rufitela	Duellman , 1967	**24(ns)**		
Boana	***B. pulchella***	***B. albonigra***	present study	**24**	**Ag-NORs, C-bands, DAPI/CMA3**	
		B. bischoffi	Beçak, 1968 (as *Hyla multilineata*)	**24**		
			Raber et al., 2004	**24**	**Ag-NORs, C-bands**	**ter 10q**
			present study	**24**	**Ag-NORs, C-bands**	**ter 11q**
		B. caingua	present study	**24**	**Ag-NORs, C-bands, DAPI/CMA3**	**int 12q**
		B. callipleura	Duellman et al., 1997	**24(ns)**		
		B. cipoensis	present study	**24**	**Ag-NORs, C-bands, DAPI/CMA3, FISH(rDNA)**	**ter 1**
		B. cordobae	Baraquet et al., 2013	**24**	**C-bands**	
			present study	**24**	**Ag-NORs, C-bands, DAPI/CMA3**	**ter 11q**
		B. curupi	Ananias et al., 2004 (as *Hyla* cf. *semiguttata*)	**24**	**Ag-NORs, C-bands**	**ter 1**
			present study	**24**	**Ag-NORs, C-bands, DAPI/CMA3**	**ter 1**
		B. guentheri	Raber et al., 2004	**24**	**Ag-NORs, C-bands**	**ter 10q**
		B. joaquini	Ananias et al., 2004 (as *Hyla semiguttata* from the localities of Cambará do Sul and São Francisco de Paula, Brazil)	**24**	**Ag-NORs, C-bands**	**ter 1**
		B. marginata	Ananias et al., 2004	**24**	**Ag-NORs, C-bands**	**int 10q**
		B. marianitae	present study	**24**	**Ag-NORs, C-bands, DAPI/CMA3**	**int 11q**
		B. polytaenia	Rabello, 1970	**24**		
			Rabello et al., 1971	**24**		
			Bogart, 1973	**24**		
			Nunes and Fagundes, 2008b	**24**		
		B. prasina	Beçak, 1968 (as *Hyla pulchella prasina*)	**24**		**ter 9q(sc)**
			Baldissera Jr et al., 1993	**24**	**Ag-NORs, C-bands**	**ter 12q[3]**
		B. pulchella	Saez and Brum, 1960 (as *Hyla raddiana raddiana*)	**24**		
			Bogart, 1973	**24**		
			Baraquet el al 2013	**24**	**C-bands**	
			present study	**24**	**Ag-NORs, C-bands, DAPI/CMA3**	**ter 12q**
		B. riojana	present study	**24**	**Ag-NORs, C-bands, DAPI/CMA3, FISH(rDNA)**	**int 11q**
		B. semiguttata	Ananias et al., 2004	**24**	**Ag-NORs, C-bands**	**ter 1**
		B. stellae	present study	**24**	**Ag-NORs, C-bands, DAPI/CMA3, FISH(rDNA)**	**ter 1**
		B. atlantica	Carvalho et al., 2014	**24**	**Ag-NORs, C-bands**	**ter 10q + ter 12q**

			Bogart, 1973 (as *Hyla granosa*)	**24**		
		B. cinerascens	Mattos et al., 2014	**24**	**Ag-NORs, C-bands, FISH(tel) (ns)**	**int 11q**
	B. punctata		present study	**24**	**Ag-NORs, C-bands, DAPI/CMA3, FISH (rDNA)**	**int 11q**
			Bogart and Bogart, 1971	**24**		
		B. punctata	Bogart, 1973	**24**		
			Anderson, 1991	**24**	**C-bands(ns)**	
			present study	**24**	**Ag-NORs, C-bands, DAPI/CMA3, FISH (rDNA)**	**ter 11q**
			Mattos et al., 2014	**24**	**Ag-NORs, C-bands, FISH (tel)**	**ter 11q**
		B. boans	Schmid and Steinlein, 2016a	**24**	**Ag-NORs, C-bands, DAPI/DA/Myt, QM, H, FISH(tel)**	**int 7q**
Boana			Schmid and Steinlein, 2016b	**24**	**Ag-NORs, C-bands, DAPI/DA/Myt, QM, H, 5-MeC**	**int 7q**
			present study	**24**	**Ag-NORs, C-bands, DAPI/CMA3, FISH (rDNA)**	**int 7q**
		B. geographica	Mattos et al., 2014	**24**	**Ag-NORs, C-bands, FISH (tel)**	**cent 1**
	B. semilineata	***B. pombali***	Carvalho et al., 2014	**24**	**Ag-NORs, C-bands**	**int 7q**
		B. semilineata	Nunes and Fagundes, 2008a	**24**	**Ag-NORs, FISH(rDNA)**	**int 7q**
			Carvalho et al. 2014	**24**	**Ag-NORs, C-bands**	**int 7q**
		B.* *cf. semilineata	present study	**24**	**Ag-NORs, C-bands, DAPI/CMA3, FISH (rDNA)**	**int 7q**
		B. wavrini	Mattos et al., 2014	**24**	**Ag-NORs, C-bands, FISH (tel)**	**ter 11q**
			present study	**24**	**Ag-NORs, C-bands, DAPI/CMA3, FISH (rDNA)**	**ter 11q**
	H. larinopygion	***H. larinopygion***	present study	**24 (*)**	**Ag-NORs, C-bands, DAPI/CMA3, FISH (rDNA)**	**ter 1**
Hyloscirtus	***H. bogotensis***	***H. alytolylax***	present study	**20 (*,**)**	**Ag-NORs, C-bands, DAPI/CMA3**	**per 4p**
		H. palmeri	present study	**24**	**Ag-NORs, C-bands, DAPI/CMA3, FISH (rDNA)**	**ter 4q**
	H. armatus	***H. armatus***	Duellman et al., 1997	**24(ns)**		

(1) Polymorphic NORs. Six of eight specimens showed heteromorphic terminal NORs on one of the homologs of pairs 1p and 7q.
(2) Additional Ag-marks were observed on ter 10q for the species, although FISH by using a rDNA probe, confirmed no signal on 10q.
(3) Polymorphic Ag-NOR. Four of five specimens showed additional terminal Ag-NORs on one or both chromosomes 9q.
(4) Inferred from DAPI-/CMA3+ marks.
(ns) Image not shown in the original publication.
(sc) Inferred exclusively from secondary constrictions.
(B) B-chromosome.
FISH Fluorescent in situ hybridization.
GISH Genomic in situ hybridization.
rDNA Ribosomal DNA probe.
tel Telomeric DNA probe.
B-probe. Chromosome probe obtained by microdissection and amplification of the B chromosome of *Boana albopunctata.*
QM Quinacrine Mustard Fluorescence.
H Hoechst 33258 fluorescence.
5-Mec Inmunofluorescence anti body for detecting 5-Methylcytosine rich heterochromatin.
(*) Only two species with a pair a telocentric chromosomes.
(**) Heteromorphic XX/XY sex chromosomes. The centromere of the Y chromosome is DAPI+/CMA3-, whereas in the X is DAPI-/CMA3+.

References list

1. Ananias F, Garcia PCA, Recco -Pimentel SM. Conserved karyotypes in the *Hyla pukhella* species group (Anura, Hylidae). Brain. 2004; 140(1):42–8.
2. Anderson K. Chromosome evolution in Holartic *Hyla* treefrogs. In: Green DM, Sessions SK. Amphibian Cytogenetics and Evolution. San Diego, Academic Press. 1991; 299–331.
3. Baldissera JR FA, Oliveira PSL, Kasahara S. Cytogenetic of four brazilian *Hyla* species (Amphibia – Anura) and descriotion of a case with supernumerary chromosome. Rev Bras Genética. 1993;16(2): 335–45.
4. Baraquet M, Salas NE, Martino AL. C-banding patterns and meiotic behavior in *Hypsiboas pulchellus* and *H. Cordobae* (anura, hylidae). BAG - J Basic Appl Genet. 2013; 24(1): 32–9.
5. Beçak ML. Chromosomal Analysis of Eighteen Species of Anura. Caryologia. 1968; 21(3): 191–208.
6. Bogart JP, Bogart JE. Genetic compatibility experiments between some south American anuran amphibians. Herpetolog. 1971; 27:229–235.
7. Bogart JP. Evolution of anuran karyotypes. En: Vial JL (Ed.) Evolutionary Biology of the Anurans. University of Missouri Press, USA. 1973; Pp. 337–349.
8. Carvalho KA, Garcia PCA, Recco-Pimentel SM **(a)**. NOR Dispersion, Telomeric Sequence Detection in Centromeric Regions and Meiotic Multivalent Configurations in Species of the *Aplastodiscus albofrenatus* Group (Anura, Hylidae). Cytogenet Genome Res.
9. Carvalho KA, Garcia PCA, Recco-Pimentel SM, Garcia PCA **(b)**. Cytogenetic comparison of tree frogs of the genus *Aplastodiscus* and the *Hypsiboas faber* group (Anura, Hylidae). Genetics and Molecular Research. 2009; 8(4):1498–508.
10. Carvalho MA, Rodrigues MT, Siqueira S, Garcia C. Dynamics of chromosomal evolution in the genus *Hypsiboas* (Anura: Hylidae). Genet Mol Res. 2014;13(3):7826–38.
11. Catroli GF, Faivovich J, Haddad CFB, Kasahara S. Conserved Karyotypes in Cophomantini: Cytogenetic Analysis of 12 Species from 3 Species Groups of *Bokermannohyla* (Amphibia: Anura: Hylidae). J Herpetol. 2011; 45(1):120–8.
12. Oliveira HHP, Souza CCN, Ribeiro CL, Bastos RP, Da Cruz AD, Silva DM. Citogenética comparativa das famílias Leptodactylidae e Hylidae do cerrado goiano. Estudos. 2012; 339(2):123–31.
13. Duellman WE. Additional studies of chromosomes of anuran amphibians. Sytematic Zoology. 1967; 16: 38–43.
14. Duellman WE, Cole CJ. Studies of Chromosomes of Some Anuran Amphibians (Hylidae and Centrolenidae). Systematic Zoology. 1965; 14(2) 139–143.
15. Duellman WE, De la Riva I, Wild ER. Frogs of the *Hyla armata* and *Hyla pulchella* group in the Andes of South America, with definitions and analyses of phylogenetic relationships of Andean group of *Hyla* . Scientific Papers. Natural History Museum, University of Kansas. 1997; 3: 1–41.
16. Ferro JM, Marti D, Bidau C, Suárez P, Nagamachi C, Pieczarka JC, et al. B chromosomes in the tree frog *Hypsiboas albopunctatus* (Anura: Hylidae). Herpetologica. 2012; 68(4):482–90.
17. Gruber SL, Zina J, Narimatsu H, Haddad CFB, Kasahara S. Comparative karyotype analysis and chromosome evolution in the genus *Aplastodiscus* (Cophomantini, Hylinae, Hylidae). BMC Genet. 2012; 13(1):28.
18. Gruber SL, Haddad CFB, Kasahara S. Chromosome banding in three species of Hypsiboas (Hylidae, Hylinae), with special reference to a new case of B-chromosome in anuran frogs and to the reduction of the diploid number of 2n = 24 to 2n = 22 in the genus. Genetica.
19. Gruber SL, Diniz D, Sobrinho-Scudeler PE, Foresti F, Haddad CFB, Kasahara S. Possible interspecific origin of the B chromosome of *Hypsiboas albopunctatus* (Spix, 1824) (Anura, Hylidae), revealed by microdissection, chromosome painting, and reverse hybridisation.
20. León PE. Report of the chromosome numbers of some Costa Rican anurans. Revista de Biologia Tropical. 1970; 17: 119–124.
21. Mattos TL, Coelho A, Schneider CE, Telles DOC, Menin M, Gross MC. Karyotypic diversity in seven Amazonian anurans in the genus Hypsiboas (family Hylidae). BMC Genet. 2014; 15(1):43.
22. Nunes RRA, Fagundes V. Patterns of ribosomal DNA distribution in hylid frogs from the *Hypsiboas faber* and *H. semilineatus* species groups. Sociedade Brasileira de Genética. 2008; 31(4): 982–987.
23. Nunes RRA, Fagundes V. Cariótipos de oito espécies de anfíbios das subfamílias Hylinae e Phyllomedusinae (Anura, Hylidae) do Espírito Santo, Brasil Bol. Mus. Biol. Mello Leitã (N. Sér.). 2008; 23: 21–36.
24. Rabello MN, Beçak ML, Beçak W. Contribução a Citotaxonomía da Família Hylidae. Arquivos do Museu Nacional. 1971; 50: 285–286.
25. Rabello MN. Chromosomal studies in Brazilian anurans. Caryologia. 1970; 23(1): 45–59.
26. Raber SC, Carvalho KA, Garcia PCA, Vinciprova G, Recco-Pimentel SM. Chromosomal characterization of *Hyla bischoffi* and *Hyla guentheri* (Anura, Hylidae). Phyllomedusa. 2004; 3:43–9.
27. Saez FA, Brum NB. Chromosomes of South American Amphibians. Nature. 1960; 4717: 945.
28. Schmid M, Steinlein C **(a)**. Chromosome Banding in Amphibia. XXXIV. Intrachromosomal Telomeric DNA Sequences in Anura. Cytogenet Genome Res. 2016; 148(2–3):211–26.
29. Schmid M, Steinlein C **(b)**. Chromosome Banding in Amphibia. XXXIII. Demonstration of 5-Methylcytosine-Rich Heterochromatin in Anura. Cytogenet Genome Res. 2016; 148(1): 35–43.
