# Supplementary material for: Chromosome evolution in Cophomantini (Amphibia, Anura, Hylinae)
Source: PLoS One. 2018 Feb 14;13(2):e0192861. doi: 10.1371/journal.pone.0192861 (PMC5812657; doi:10.1371/journal.pone.0192861)
Supplement: S2 Table — (a) B. albopunctata group; (b) B. faber group; (c) B. pellucens group; (d) B. pulchella group; (e) B. punctata group; (f) B. semilineata group. (B) B chromosome in B. leucocheila. Chromosome percentage relative to the haploid set (Chromosome Mophology) Centromeric Index ± Standard Deviation. m: metacentric; sm: submetacentric; st: subtelocentric; t: telocentric. (PDF) [file pone.0192861.s003.pdf]

S2 Table. Chromosome measurements of 25 species of *Boana* and 3 of *Hyloscirtus*. (a) *B. albopunctata* group; (b) *B. faber* group; (c) *B. pellucens* group; (d) *B. pulchella* group; (e) *B. punctata* group; (f) *B. semilineata* group. [(B)] B chromosome in *B. leucocheila*. Chromosome percentage relative to the haploid set. Centromeric Index ± Standard Deviation. m: metacentric; sm: submetacentric; st: subtelocentric; t: telocentric.

Species of Boana	***Chromosome Pair***											
	1	***2***	***3***	***4***	***5***	***6***	***7***	***8***	***9***	***10***	***11***	***12***
***B. almendarizae* (a)**	14.99 **(m)**<br>.48 ± .02	12.54 **(m)**<br>.40 ± .01	10.77 **(sm)**<br>.31 ± .02	11.39 **(sm)**<br>.25 ± .02	9.68 **(sm)**<br>.33 ± .03	8.06 **(sm)**<br>.27 ± .01	6.30 **(sm)**<br>.37 ± .02	6.36 **(m)**<br>.42 ± .03	6.02 **(sm)**<br>.36 ± .03	5.27 **(m)**<br>.46 ± .05	4.64 **(m)**<br>.42 ± .02	3.99 **(m)**<br>.45 ± .01
***B. calcarata* (a)**	17.4 **(m)**<br>.47 ± .01	12.76 **(m)**<br>.40 ± .01	10.84 **(sm)**<br>.33 ± .02	10.35 **(st)**<br>.24 ± .02	8.78 **(sm)**<br>.33 ± .02	8.12 **(sm)**<br>.34 ± .01	6.48 **(m)**<br>.38 ± .03	5.63 **(m)**<br>.44 ± .01	5.85 **(m)**<br>.42 ± .01	5.08 **(m)**<br>.44 ± .03	5.04 **(m)**<br>.40 ± .00	3.67 **(m)**<br>.45 ± .01
***B.* cf. *alfaroi* (a)**	15.92 **(m)**<br>.48 ± .01	12.79 **(m)**<br>.39 ± .03	11.41 **(sm)**<br>.36 ± .02	11.07 **(sm)**<br>.26 ± .03	10.29 **(sm)**<br>.31 ± .02	8.06 **(sm)**<br>.29 ± .05	6.86 **(sm)**<br>.36 ± .04	6.68 **(m)**<br>.48 ± .01	6.12 **(m)**<br>.47 ± .02	5.58 **(sm)**<br>.36 ± .04	5.38 **(m)**<br>.45 ± .03	
***B. heilprini* (a)**	14.02 **(m)**<br>.49 ± .00	13.57 **(m)**<br>.41 ± .01	11.73 **(sm)**<br>.32 ± .00	11.37 **(sm)**<br>.25 ± .00	10.4 **(m)**<br>.39 ± .04	9.15 **(sm)**<br>.30 ± .00	7.12 **(sm)**<br>.32 ± .01	5.43 **(m)**<br>.46 ± .01	5.13 **(sm)**<br>.30 ± .01	4.78 **(m)**<br>.45 ± .01	4.05 **(m)**<br>.45 ± .00	3.25 **(sm)**<br>.30 ± .01
***B.* cf. *lanciformis* (a)**	15.37 **(m)**<br>.49 ± .01	13.34 **(m)**<br>.40 ± .01	11.65 **(sm)**<br>.37 ± .02	11.35 **(st)**<br>.22 ± .01	10.41 **(sm)**<br>.30 ± .02	8.27 **(st)**<br>.24 ± .01	6.02 **(sm)**<br>.34 ± .02	5.75 **(m)**<br>.39 ± .02	5.35 **(m)**<br>.46 ± .01	5.06 **(sm)**<br>.45 ± .02	3.99 **(sm)**<br>.36 ± .01	3.44 **(m)**<br>.44 ± .01
***B. leucocheila* (a)**	18.57 **(m)**<br>.47 ± .02	13.20 **(m)**<br>.41 ± .02	11.55 **(sm)**<br>.33 ± .03	10.86 **(sm)**<br>.25 ± .03	9.93 **(sm)**<br>.30 ± .03	8.23 **(sm)**<br>.26 ± .02	6.02 **(m)**<br>.38 ± .04	6.03 **(m)**<br>.47 ± .02	5.36 **(m)**<br>.44 ± .03	5.11 **(sm)**<br>.37 ± .03	4.57 **(m)**<br>.40 ± .04	3.11 **(m)**[(B)]<br>.46 ± .01
***B. multifasciata* (a)**	17.32 **(m)**<br>.48 ± .01	13.10 **(m)**<br>.40 ± .02	11.53 **(sm)**<br>.34 ± .02	10.52 **(st)**<br>.24 ± .01	9.58 **(sm)**<br>.32 ± .02	8.20 **(st)**<br>.24 ± .01	6.59 **(sm)**<br>.36 ± .01	6.74 **(m)**<br>.47 ± .02	5.89 **(m)**<br>.45 ± .02	5.47 **(sm)**<br>.34 ± .03	5.07 **(m)**<br>.44 ± .01	
***B. raniceps* (a)**	15.73 **(m)**<br>.48 ± .02	12.50 **(m)**<br>.41 ± .01	11.24 **(sm)**<br>.35 ± .01	10.23 **(st)**<br>.24 ± .01	9.95 **(sm)**<br>.34 ± .02	8.52 **(sm)**<br>.33 ± .02	6.59 **(m)**<br>.39 ± .02	6.07 **(m)**<br>.47 ± .02	5.79 **(sm)**<br>.33 ± .01	4.86 **(m)**<br>.44 ± .02	4.65 **(m)**<br>.45 ± .02	3.87 **(sm)**<br>.36 ± .01
***B. faber* (b)**	14.30 **(m)**<br>.47 ± .02	12.80 **(m)**<br>.42 ± .01	10.97 **(sm)**<br>.33 ± .01	11.23 **(st)**<br>.24 ± .01	10.16 **(sm)**<br>.37 ± .02	7.81 **(st)**<br>.23 ± .01	7.12 **(st)**<br>.23 ± .01	6.42 **(m)**<br>.45 ± .02	5.70 **(sm)**<br>.45 ± .01	5.73 **(sm)**<br>.36 ± .01	4.43 **(m)**<br>.39 ± .01	3.33 **(m)**<br>.43 ± .03
***B. pellucens* (c)**	15.74 **(m)**<br>.48 ± .01	12.84 **(m)**<br>.40 ± .03	11.53 **(sm)**<br>.31 ± .02	10.36 **(sm)**<br>.35 ± .01	9.49 **(sm)**<br>.35 ± .02	7.91 **(sm)**<br>.33 ± .02	6.85 **(m)**<br>.45 ± .02	6.31 **(m)**<br>.48 ± .03	5.39 **(sm)**<br>.33 ± .02	5.28 **(m)**<br>.46 ± .01	4.73 **(sm)**<br>.33 ± .02	3.57 **(m)**<br>.46 ± .02
***B. albonigra* (d)**	15.75 **(m)**<br>.48 ± .00	12.52 **(m)**<br>.40 ± .02	11.30 **(sm)**<br>.35 ± .01	10.28 **(st)**<br>.24 ± .01	9.67 **(sm)**<br>.35 ± .03	7.85 **(st)**<br>.25 ± .01	6.64 **(sm)**<br>.34 ± .01	5.99 **(sm)**<br>.37 ± .02	6.06 **(m)**<br>.47 ± .03	5.21 **(m)**<br>.43 ± .02	4.27 **(m)**<br>.43 ± .01	3.11 **(m)**<br>.48 ± .00
***B. caingua* (d)**	17.56 **(m)**<br>.47 ± .01	12.55 **(m)**<br>.40 ± .01	10.90 **(sm)**<br>.34 ± .01	10.24 **(st)**<br>.23 ± .00	9.58 **(sm)**<br>.32 ± .02	7.61 **(st)**<br>.22 ± .01	6.52 **(sm)**<br>.33 ± .01	5.77 **(m)**<br>.38 ± .01	5.65 **(m)**<br>.46 ± .02	5.14 **(m)**<br>.46 ± .01	4.45 **(m)**<br>.45 ± .01	4.03 **(m)**<br>.47 ± .01
***B. cipoensis* (d)**	17.04 **(m)**<br>.48 ± .01	12.85 **(m)**<br>.39 ± .02	11.24 **(sm)**<br>.31 ± .01	10.35 **(st)**<br>.23 ± .01	9.76 **(sm)**<br>.30 ± .02	8.15 **(st)**<br>.24 ± .01	6.49 **(sm)**<br>.33 ± .02	5.70 **(sm)**<br>.37 ± .02	5.34 **(m)**<br>.44 ± .01	5.19 **(m)**<br>.44 ± .01	4.11 **(m)**<br>.45 ± .02	3.77 **(m)**<br>.45 ± .02
***B. cordobae* (d)**	17.32 **(m)**<br>.48 ± .01	12.76 **(m)**<br>.39 ± .01	10.96 **(sm)**<br>.32 ± .01	11.01 **(st)**<br>.24 ± .01	9.76 **(sm)**<br>.32 ± .01	7.70 **(st)**<br>.24 ± .01	6.03 **(sm)**<br>.35 ± .02	5.72 **(m)**<br>.41 ± .01	5.48 **(m)**<br>.46 ± .03	4.88 **(m)**<br>.45 ± .01	4.44 **(m)**<br>.46 ± .03	3.94 **(m)**<br>.46 ± .02
***B. curupi* (d)**	18.82 **(m)**<br>.49 ± .01	13.13 **(m)**<br>.39 ± .00	11.34 **(sm)**<br>.32 ± .01	10.81 **(st)**<br>.22 ± .01	10.00 **(sm)**<br>.31 ± .01	8.07 **(st)**<br>.24 ± .01	5.98 **(sm)**<br>.33 ± .01	5.34 **(sm)**<br>.36 ± .02	5.23 **(m)**<br>.47 ± .02	4.43 **(m)**<br>.46 ± .00	3.74 **(m)**<br>.47 ± .00	3.10 **(m)**<br>.45 ± .01
***B. bischoffi* (d)**	16.44 **(m)**<br>.47 ± .03	13.16 **(m)**<br>.39 ± .01	11.66 **(sm)**<br>.33 ± .01	10.91 **(st)**<br>.22 ± .01	10.42 **(sm)**<br>.28 ± .02	8.18 **(st)**<br>.24 ± .01	5.89 **(sm)**<br>.32 ± .02	5.68 **(sm)**<br>.32 ± .01	5.67 **(m)**<br>.47 ± .02	4.29 **(m)**<br>.43 ± .04	4.28 **(m)**<br>.45 ± .01	3.43 **(m)**<br>.47 ± .02
***B. marianitae* (d)**	13.98 **(m)**<br>.46 ± .02	12.46 **(m)**<br>.39 ± .00	10.92 **(sm)**<br>.35 ± .00	10.72 **(st)**<br>.23 ± .01	9.83 **(sm)**<br>.33 ± .01	7.84 **(st)**<br>.24 ± .00	6.69 **(sm)**<br>.33 ± .01	6.32 **(sm)**<br>.36 ± .00	6.34 **(m)**<br>.45 ± .01	5.43 **(m)**<br>.45 ± .02	5.10 **(m)**<br>.44 ± .00	4.36 **(m)**<br>.45 ± .00
***B. pulchella* (d)**	16.94 **(m)**<br>.47 ± .02	13.16 **(m)**<br>.41 ± .03	10.95 **(sm)**<br>.36 ± .01	10.66 **(st)**<br>.22 ± .01	9.76 **(sm)**<br>.33 ± .01	7.76 **(st)**<br>.20 ± .00	6.06 **(sm)**<br>.32 ± .02	5.66 **(m)**<br>.40 ± .03	5.35 **(m)**<br>.45 ± .02	5.08 **(m)**<br>.45 ± .01	4.41 **(m)**<br>.46 ± .01	4.21 **(m)**<br>.46 ± .02
***B. riojana* (d)**	16.68 **(m)**<br>.47 ± .02	13.21 **(m)**<br>.39 ± .00	11.32 **(sm)**<br>.33 ± .01	10.74 **(st)**<br>.22 ± .01	10.14 **(sm)**<br>.32 ± .02	7.92 **(st)**<br>.22 ± .02	6.28 **(sm)**<br>.32 ± .02	5.50 **(sm)**<br>.34 ± .02	5.57 **(m)**<br>.45 ± .02	4.73 **(m)**<br>.44 ± .03	4.51 **(m)**<br>.45 ± .02	3.41 **(m)**<br>.46 ± .01
***B. stellae* (d)**	17.53 **(m)**<br>.47 ± .01	12.25 **(m)**<br>.41 ± .02	10.98 **(sm)**<br>.32 ± .02	10.43 **(st)**<br>.23 ± .01	9.79 **(sm)**<br>.31 ± .02	8.24 **(st)**<br>.23 ± .01	6.48 **(sm)**<br>.31 ± .03	5.79 **(sm)**<br>.34 ± .01	5.51 **(m)**<br>.40 ± .02	4.78 **(m)**<br>.46 ± .02	4.39 **(m)**<br>.45 ± .03	3.83 **(m)**<br>.45 ± .02
***B. cinerascens* (e)**	18.31 **(m)**<br>.48 ± .01	12.87 **(m)**<br>.38 ± .02	11.63 **(sm)**<br>.34 ± .02	11.27 **(st)**<br>.24 ± .01	10.08 **(sm)**<br>.31 ± .02	8.19 **(st)**<br>.20 ± .02	5.80 **(sm)**<br>.29 ± .02	5.64 **(m)**<br>.45 ± .04	5.14 **(m)**<br>.40 ± .04	4.74 **(sm)**<br>.37 ± .02	3.37 **(m)**<br>.47 ± .02	2.96 **(m)**<br>.48 ± .01
***B. punctata* (e)**	17.43 **(m)**<br>.48 ± .01	12.95 **(m)**<br>.40 ± .01	10.81 **(sm)**<br>.35 ± .01	10.56 **(sm)**<br>.26 ± .02	10.29 **(sm)**<br>.37 ± .02	7.72 **(st)**<br>.24 ± .01	6.13 **(sm)**<br>.27 ± .02	5.58 **(m)**<br>.45 ± .01	5.27 **(m)**<br>.46 ± .02	4.91 **(sm)**<br>.31 ± .03	**A** 2.28**(m)** .46 ± .01<br>**B** 2.04**(sm)** .32 ± .02	4.03 **(sm)**<br>.35 ± .01
***B. boans* (f)**	15.15 **(m)**<br>.48 ± .01	13.00 **(m)**<br>.39 ± .01	10.33 **(sm)**<br>.34 ± .02	10.01 **(st)**<br>.23 ± .01	9.72 **(sm)**<br>.36 ± .01	7.70 **(sm)**<br>.25 ± .02	6.36 **(sm)**<br>.31 ± .03	**A** 3.30**(sm)** .34 ± .02<br>**B** 3.84**(m)** .41 ± .01	5.57 **(m)**<br>.44 ± .02	5.43 **(m)**<br>.45 ± .02	5.02 **(m)**<br>.44 ± .02	4.58 **(m)**<br>.45 ± .02
***B.* cf. *semilineata* (f)**	14.50 **(m)**<br>.48 ± .01	11.27 **(m)**<br>.40 ± .01	10.13 **(sm)**<br>.34 ± .01	10.55 **(st)**<br>.24 ± .01	9.48 **(sm)**<br>.34 ± .01	8.56 **(sm)**<br>.28 ± .02	7.63 **(sm)**<br>.37 ± .02	6.92 **(sm)**<br>.29 ± .02	6.28 **(sm)**<br>.33 ± .02	5.33 **(m)**<br>.39 ± .02	5.25 **(m)**<br>.45 ± .01	4.20 **(m)**<br>.46 ± .01
***B. wavrini* (f)**	17.58 **(m)**<br>.48 ± .01	12.58 **(m)**<br>.40 ± .02	10.58 **(sm)**<br>.34 ± .03	10.43 **(st)**<br>.23 ± .01	9.62 **(sm)**<br>.25 ± .03	7.75 **(sm)**<br>.25 ± .02	6.57 **(sm)**<br>.30 ± .02	5.89 **(sm)**<br>.36 ± .03	5.42 **(m)**<br>.44 ± .04	4.89 **(m)**<br>.46 ± .01	4.54 **(m)**<br>.44 ± .01	4.13 **(m)**<br>.46 ± .01

Species of Hyloscirtus	***Chromosome Pair***											
	1	***2***	***3***	***4***	***5***	***6***	***7***	***8***	***9***	***10***	***11***	***12***
H. larinopygion	18.56 **(m)**<br>.46 ± .01	13.17 **(m)**<br>.40 ± .01	11.66 **(sm)**<br>.32 ± .01	10.77 **(st)**<br>.24 ± .00	10.3 **(sm)**<br>.26 ± .01	8.00 **(sm)**<br>.28 ± .01	6.25 **(sm)**<br>.35 ± .02	5.04 **(m)**<br>.43 ± .01	4.66 **(m)**<br>.43 ± .02	4.32 **(m)**<br>.46 ± .02	3.92 **(m)**<br>.47 ± .01	3.35 **(t)**<br>.00 ± .00
H. alytolylax	19.34 **(m)**<br>.42 ± .01	**X** 6.80**(m)** .40 ± .01<br>**Y** 6.15**(m)** .43 ± .02	11.79 **(sm)**<br>.36 ± .02	11.95 **(sm)**<br>.31 ± .02	10.37 **(sm)**<br>.34 ± .02	10.03 **(m)**<br>.45 ± .01	6.91 **(t)**<br>.00 ± .00	6.65 **(m)**<br>.41 ± .02	5.49 **(m)**<br>.40 ± .01	4.38 **(m)**<br>.47 ± .02		
H. palmeri	14.65 **(m)**<br>.48 ± .01	11.64 **(m)**<br>.39 ± .02	10.32 **(sm)**<br>.35 ± .02	11.04 **(st)**<br>.24 ± .01	9.49 **(sm)**<br>.36 ± .01	9.12 **(sm)**<br>.36 ± .01	6.62 **(m)**<br>.38 ± .00	6.08 **(sm)**<br>.33 ± .02	6.10 **(m)**<br>.44 ± .02	5.54 **(m)**<br>.46 ± .01	5.03 **(m)**<br>.45 ± .01	4.36 **(m)**<br>.47 ± .01
