# Supplementary material for: Chromosome evolution in Cophomantini (Amphibia, Anura, Hylinae)
Source: PLoS One. 2018 Feb 14;13(2):e0192861. doi: 10.1371/journal.pone.0192861 (PMC5812657; doi:10.1371/journal.pone.0192861)
Supplement: S1 Fig — (PDF) [file pone.0192861.s004.pdf]

S1 Fig. Optimization of the basic number (X) and the position of NORs (NORs) in Cophomantini on the phylogenetic hypothesis of Duellman et al. (2016).

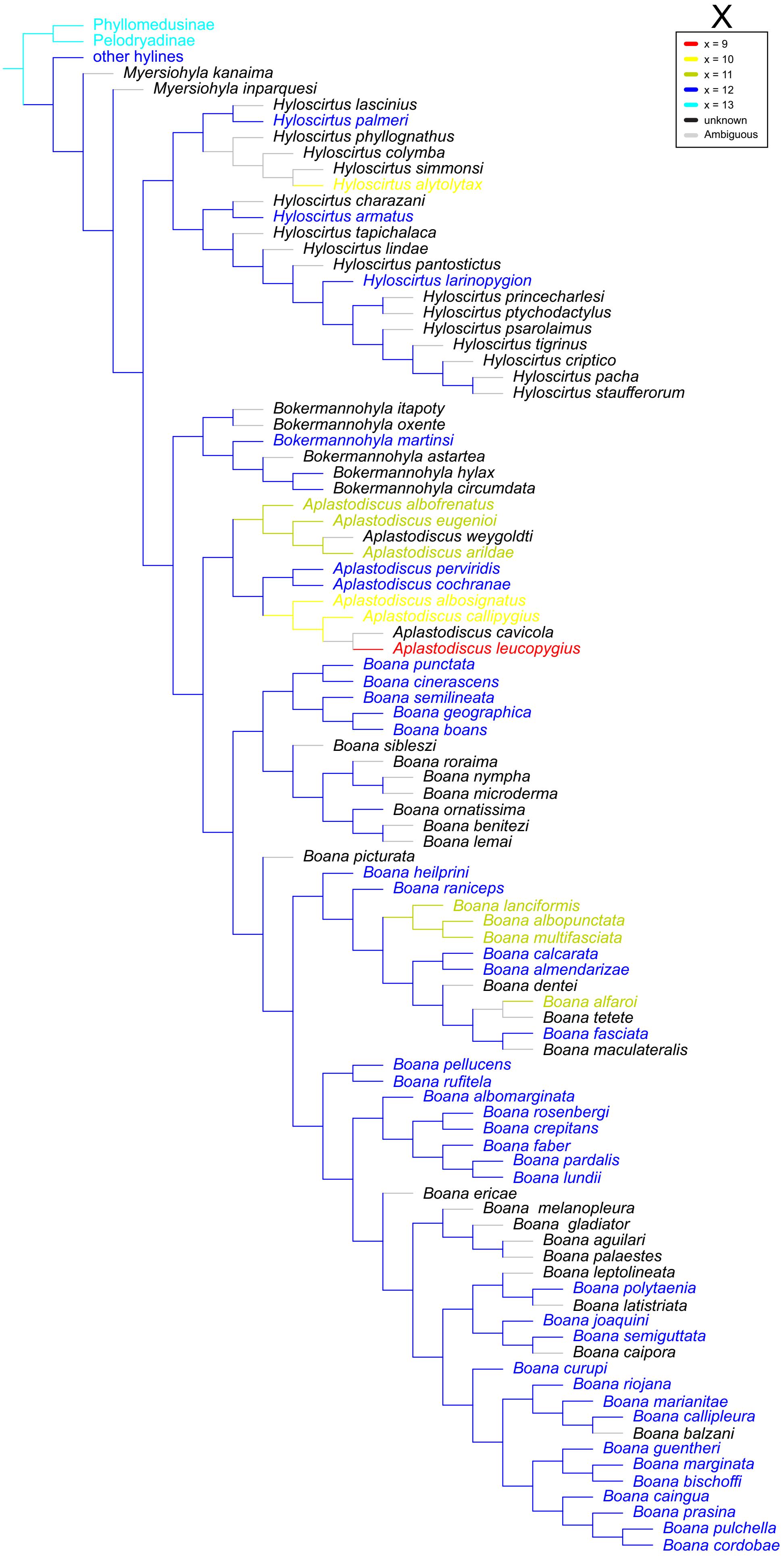
X
x = 9
x = 10
x = 11
x = 12
x = 13
unknown
Ambiguous
Phyllomedusinae
Pelodryadinae
other hylines
Myersiohyla kanaima
Myersiohyla inparquesi
Hyloscirtus lascinius
Hyloscirtus palmeri
Hyloscirtus phyllognathus
Hyloscirtus colymba
Hyloscirtus simmonsi
Hyloscirtus alytolytax
Hyloscirtus charazani
Hyloscirtus armatus
Hyloscirtus tapichalaca
Hyloscirtus lindae
Hyloscirtus pantostictus
Hyloscirtus larinopygion
Hyloscirtus princecharlesi
Hyloscirtus ptychodactylus
Hyloscirtus psarolaimus
Hyloscirtus tigrinus
Hyloscirtus criptico
Hyloscirtus pacha
Hyloscirtus staufferorum
Bokermannohyla itapoty
Bokermannohyla oxente
Bokermannohyla martinsi
Bokermannohyla astartea
Bokermannohyla hylax
Bokermannohyla circumdata
Aplastodiscus albofrenatus
Aplastodiscus eugenioi
Aplastodiscus weygoldti
Aplastodiscus arildae
Aplastodiscus perviridis
Aplastodiscus cochranae
Aplastodiscus albosignatus
Aplastodiscus callipygius
Aplastodiscus cavicola
Aplastodiscus leucopygius
Boana punctata
Boana cinerascens
Boana semilineata
Boana geographica
Boana boans
Boana sibleszi
Boana roraima
Boana nympha
Boana microderma
Boana ornatissima
Boana benitezi
Boana lemai
Boana picturata
Boana heilprini
Boana raniceps
Boana lanciformis
Boana albopunctata
Boana multifasciata
Boana calcarata
Boana almendarizae
Boana dentei
Boana alfaroi
Boana tetete
Boana fasciata
Boana maculateralis
Boana pellucens
Boana rufitela
Boana albomarginata
Boana rosenbergi
Boana crepitans
Boana faber
Boana pardalis
Boana lundii
Boana ericae
Boana melanopleura
Boana gladiator
Boana aguilari
Boana palaestes
Boana leptolineata
Boana polytaenia
Boana latistriata
Boana joaquini
Boana semiguttata
Boana caipora
Boana curupi
Boana riojana
Boana marianitae
Boana callipleura
Boana balzani
Boana guentheri
Boana marginata
Boana bischoffi
Boana caingua
Boana prasina
Boana pulchella
Boana cordobae

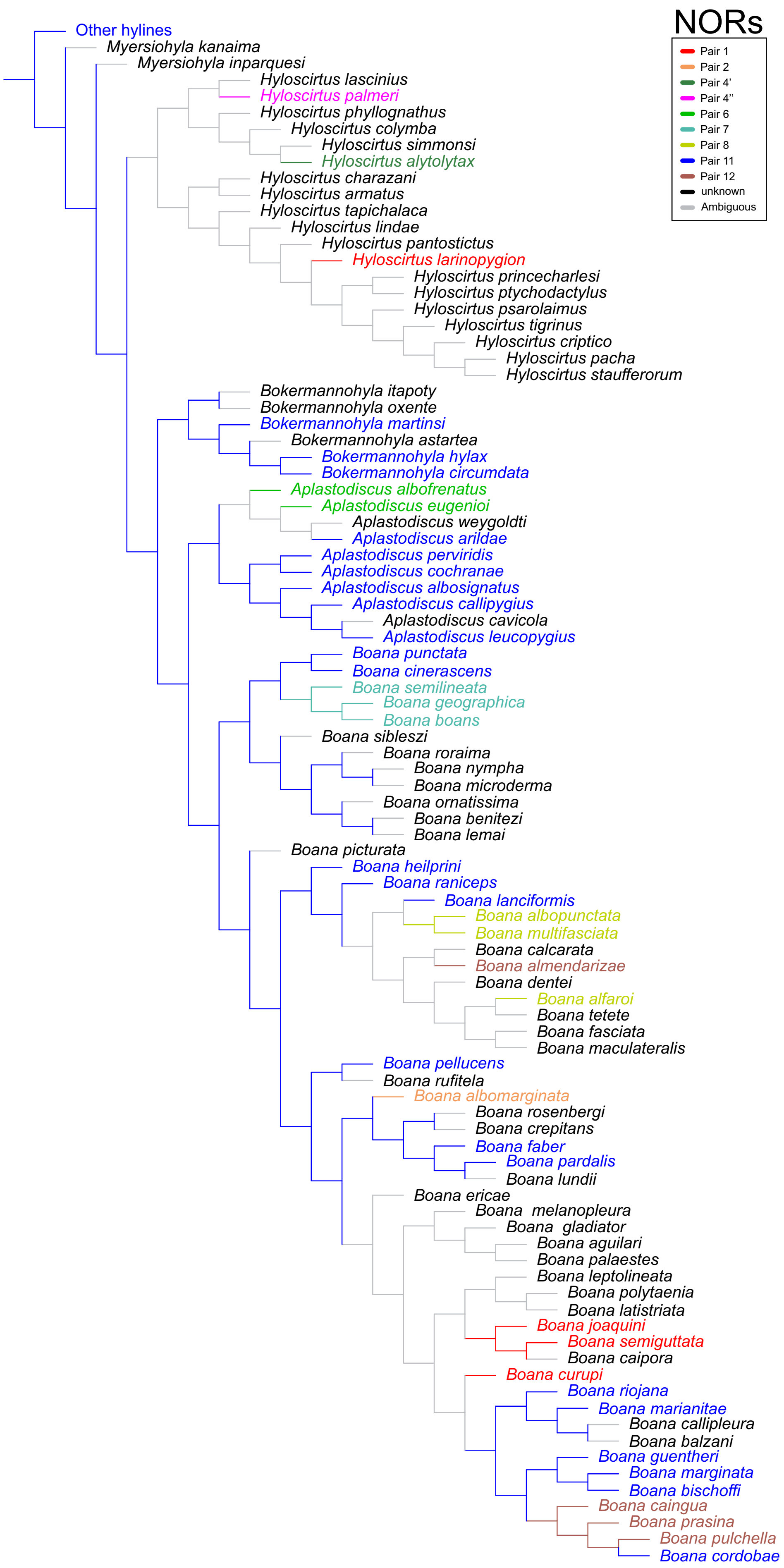
NORs
Pair 1
Pair 2
Pair 4'
Pair 4''
Pair 6
Pair 7
Pair 8
Pair 11
Pair 12
unknown
Ambiguous
Other hylines
Myersiohyla kanaima
Myersiohyla inparquesi
Hyloscirtus lascinius
Hyloscirtus palmeri
Hyloscirtus phyllognathus
Hyloscirtus colymba
Hyloscirtus simmonsi
Hyloscirtus alytolytax
Hyloscirtus charazani
Hyloscirtus armatus
Hyloscirtus tapichalaca
Hyloscirtus lindae
Hyloscirtus pantostictus
Hyloscirtus larinopygion
Hyloscirtus princecharlesi
Hyloscirtus ptychodactylus
Hyloscirtus psarolaimus
Hyloscirtus tigrinus
Hyloscirtus criptico
Hyloscirtus pacha
Hyloscirtus staufferorum
Bokermannohyla itapoty
Bokermannohyla oxente
Bokermannohyla martinsi
Bokermannohyla astartea
Bokermannohyla hylax
Bokermannohyla circumdata
Aplastodiscus albofrenatus
Aplastodiscus eugenioi
Aplastodiscus weygoldti
Aplastodiscus arildae
Aplastodiscus perviridis
Aplastodiscus cochranae
Aplastodiscus albosignatus
Aplastodiscus callipygius
Aplastodiscus cavicola
Aplastodiscus leucopygius
Boana punctata
Boana cinerascens
Boana semilineata
Boana geographica
Boana boans
Boana sibleszi
Boana roraima
Boana nympha
Boana microderma
Boana ornatissima
Boana benitezi
Boana lemai
Boana picturata
Boana heilprini
Boana raniceps
Boana lanciformis
Boana albopunctata
Boana multifasciata
Boana calcarata
Boana almendarizae
Boana dentei
Boana alfaroi
Boana tetete
Boana fasciata
Boana maculateralis
Boana pellucens
Boana rufitela
Boana albomarginata
Boana rosenbergi
Boana crepitans
Boana faber
Boana pardalis
Boana lundii
Boana ericae
Boana melanopleura
Boana gladiator
Boana aguilari
Boana palaestes
Boana leptolineata
Boana polytaenia
Boana latistriata
Boana joaquini
Boana semiguttata
Boana caipora
Boana curupi
Boana riojana
Boana marianitae
Boana callipleura
Boana balzani
Boana guentheri
Boana marginata
Boana bischoffi
Boana caingua
Boana prasina
Boana pulchella
Boana cordobae
