# Supplementary figures and images for: Chromosome evolution in Cophomantini (Amphibia, Anura, Hylinae)
Source: PLoS One. 2018 Feb 14;13(2):e0192861. doi: 10.1371/journal.pone.0192861 (PMC5812657; doi:10.1371/journal.pone.0192861)

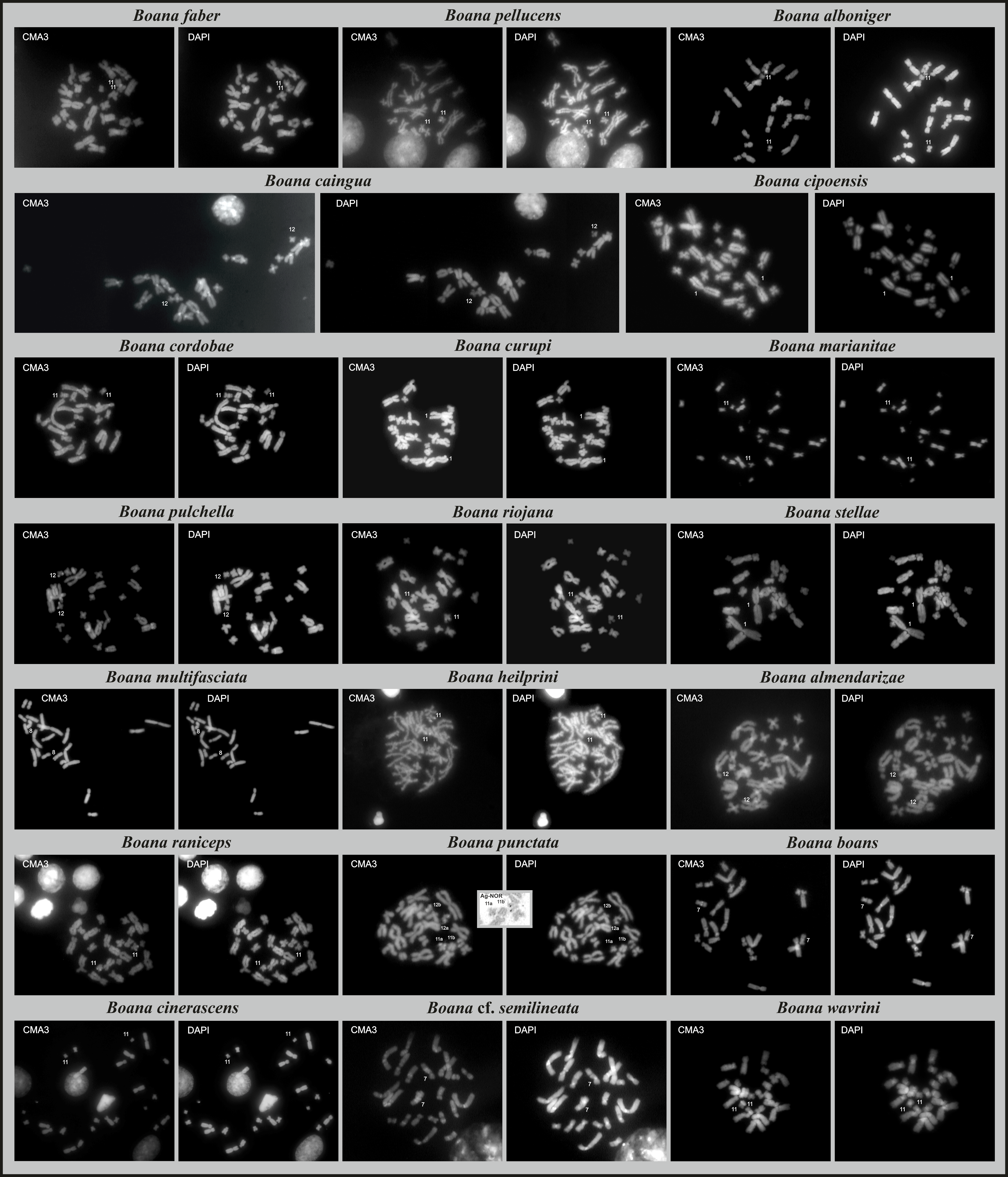

Supplement: S2 Fig — The square shows heteromorphic NOR-bearing chromosome pair in B. punctata sequentially stained by the silver impregnation technique. (TIF) [file pone.0192861.s005.tif]
